# A comprehensive approach to the design and development of a low-cost semi-automatic blood pressure monitoring device for resource-constrained

**DOI:** 10.1016/j.ohx.2026.e00843

**Published:** 2026-09-19

**Authors:** Muhammad Al-Ayyad, Bashar Al-haj Moh’d, Mohammad Hasan Shamsan, Mohammad Al-Sayed Ahmad, Mohammad Al-Takrouri, Hamza Abu Owida

**Affiliations:** a Medical Engineering Department, Al-Ahliyya Amman University, Al-Salt Road, Amman 19111, Jordan; b Research and Development Department, New Generation Electronics Co., 147, Queen Rania St, Swaileh, Amman, Jordan; c Faculty of Electrical Engineering, University Teknologi Malaysia, Johor, Malaysia

**Keywords:** Low-cost medical devices, Blood pressure monitoring, Semi-automatic systems, Arduino microcontroller, Preventive healthcare

## Abstract

Hypertension is a major global healthcare challenge, particularly for low-income individuals who often struggle to access monitoring devices. This paper presents the design of a cost-effective, semi-automatic blood pressure monitoring device aimed at improving accessibility for these patients, thereby enhancing preventive care and reducing mortality risks. The proposed design replaces an expensive electric air pump with a manual inflation bulb, while integrating a digital display for accurate pressure readings. The system comprises three key components: a robust electronic circuit, a reliable pneumatic system, and a 3D-printed housing optimized for semi-automatic operation. A notable aspect of this work is the hardware–software integration, which utilizes an inexpensive, single-core Arduino microcontroller (MCU) to achieve precise measurements. Despite relying on affordable components, the device delivers consistent and accurate results, demonstrating the efficiency of the design. The total manufacturing cost of the device is under $8, making it a highly accessible solution for resource-constrained settings. This paper provides comprehensive details of the design and implementation, enabling researchers to replicate the device and explore further improvements, particularly in software optimization and feature expansion. By addressing both affordability and accuracy, this work offers a practical and scalable solution for blood pressure monitoring, contributing to global efforts in preventive healthcare.

## Graphical abstract

**Figure:**
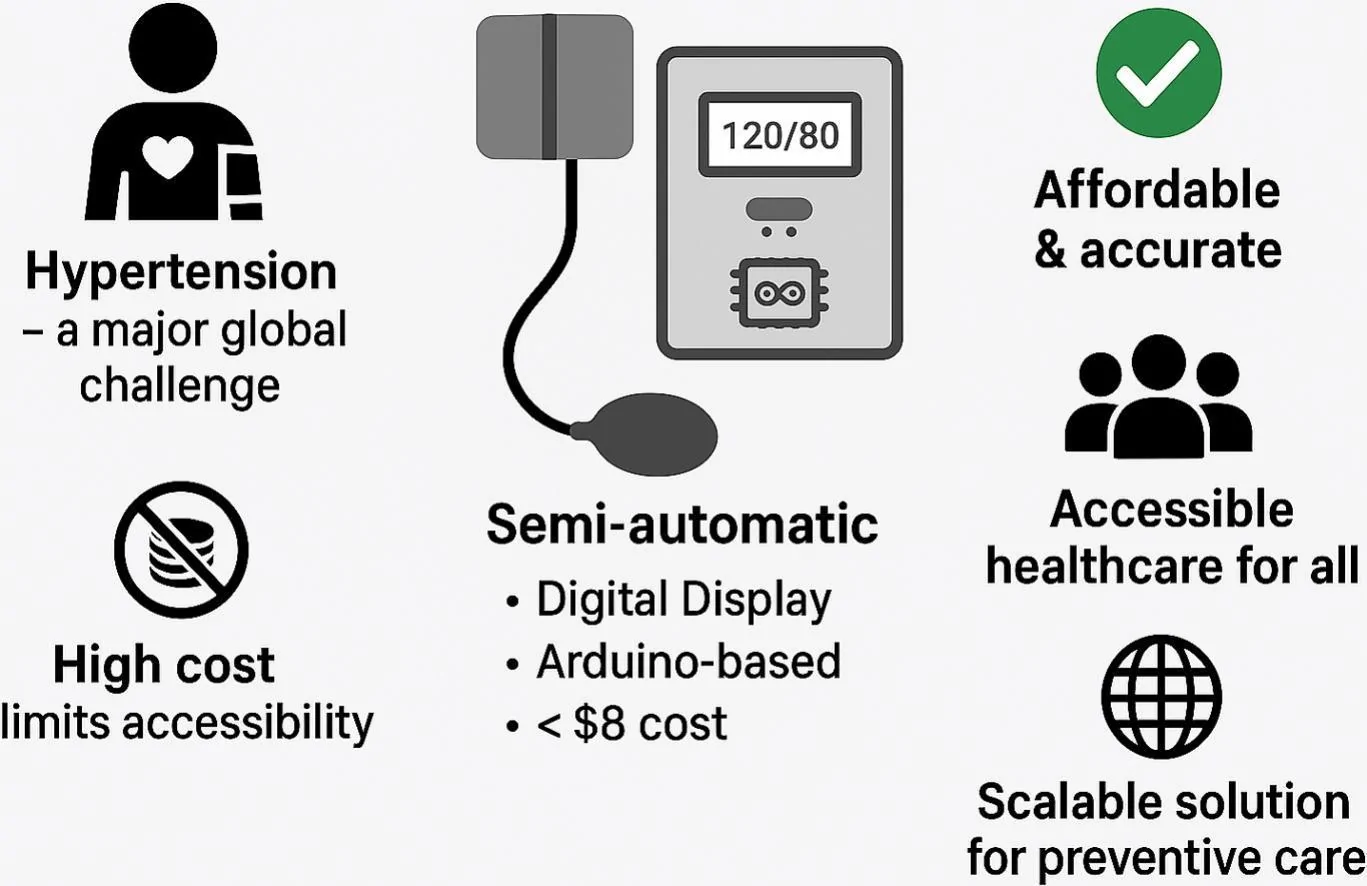

## 1 Specifications table

**Table:** 

Hardware name	*PressSure*
Subject area	• Engineering and materials science • Medical • Educational tools and open-source alternatives to existing infrastructure
Hardware type	• Field measurements and sensors • Electrical engineering and computer science • Mechanical engineering and materials science
Closest commercial analog	*“The closest commercial analog to our hardware is a standard automatic digital blood pressure monitor. However, our device significantly reduces cost by eliminating the air pump and solenoid valve, using manual inflation and a controlled leakage system instead. This makes it a viable low-cost alternative for resource-limited settings. Additionally, it offers improved accuracy compared to inexpensive digital monitors, which often suffer from calibration drift and measurement inconsistencies.”*
Open source license	MIT license
Cost of hardware	*$8 USD*
Source file repository	**10.17605/OSF.IO/4RAB3**

## 2 Hardware in context

Globally, cardiovascular diseases (CVDs) are the primary cause of mortality; elevated systolic arterial pressure is a silent killer that causes over 10 million deaths annually [1]. This is evident in nations with limited resources which have lower rates of hypertension awareness and control compared with their industrialized counterparts [2]. Regardless of gender or nationality, reducing blood pressure (BP) lowers the risk of CVD morbidity and all-cause mortality [3].

In medical and physiological practice, blood pressure is among the most valuable measurable vital signs that are monitored and investigated. Evaluation of cardiovascular (CV) status and some other aspects of cardiac function can be achieved by simply determining its maximum and minimum values during every heartbeat [4]. When this information is combined with data about other physiological factors, it becomes an even more useful diagnostic tool [5].

Early detection and accurate diagnosis are necessary for primary care patients with hypertension; this can help avoid many related fatalities. These patients also need to be screened for high blood Early detection and accurate diagnosis are necessary for primary care patients with hypertension; this can help avoid many related fatalities. These patients also need to be screened for high blood pressure and rechecked within four weeks, supplemented by effective management of hypertension [6], [7]. A condition known as “*white coat syndrome*” occurs when a patient’s blood pressure measurements in a clinical environment are noticeably higher than those obtained at home. Anxiety or stress during medical appointments is frequently linked to this syndrome [8], [9], [10].

and rechecked within four weeks, supplemented by effective management of hypertension [6], [7]. A condition known as “white coat syndrome” occurs when a patient’s blood pressure measurements in a clinical environment are noticeably higher than those obtained at home. Anxiety or stress during medical appointments is frequently linked to this syndrome [8], [9], [10].

White-coat hypertension can be avoided if measurements are obtained at the patient’s home, since these measurements have a higher correlation to 24-hour blood pressure values than do readings from clinics. According to hypertension guidelines, the average value of blood pressure readings taken at rest should be used to obtain informative reliable results [11].

Patients who self-monitor their blood pressure at home tend to feel more confident and relaxed during the measurement process, particularly when they are experiencing symptoms. Additionally, home blood pressure monitoring can potentially lead to reduced healthcare costs.

Despite its popularity, the manual auscultatory technique has many sources of user errors. Since it depends mainly on the user’s ability to identify the phases of Korotkoff sounds, the user may overestimate or underestimate systolic and diastolic blood pressure readings if they miss the exact initiation of the first Korotkoff sound and the exact disappearance of the last Korotkoff sound. Furthermore, a fast cuff deflation rate, high ambient noise, terminal digit bias, and an inaccurate manometer can also cause errors [12].

Many techniques have been developed thanks to the extensive research and development in automatic arterial pressure measurement devices, but only a small number of them have been marketed because of high development costs, which are not within everyone’s reach [8]. A study [13] found that the average price of blood pressure measuring devices across the 100 best-selling Amazon lists in 10 countries was $32. The World Health Organization (WHO), acknowledging the challenges of measuring blood pressure in low-resource environments, outlined several conditions for an effective device. The foremost requirement was accuracy. Additionally, it was suggested that the ideal device should be durable, affordable, and easy to use with minimal training.

Semi-automated blood pressure monitors, which combine affordability, accuracy, and ease of use, are an important development in the management of hypertension. They reduce the possibility of measurement errors that are frequently associated with conventional procedures and provide individuals with the power to take control of their health.

In the current landscape of blood pressure monitors, most solutions suffer from high manufacturing costs, limiting their accessibility. This paper presents a low-cost design for a semi-automated blood pressure monitor that maintains comparable performance to existing devices, reducing the production cost to under $8 while ensuring accuracy, ease of use, reliability, and durability.

## 3 Hardware description

The concept behind the proposed device is to replace relatively expensive components with more cost-effective alternatives, while maintaining ease of use, accurate measurements, and essential features. Specifically, the proposed design eliminates the use of an air pump and a solenoid valve, simplifying the system without compromising functionality. Fig. 1 presents the fundamental block diagram of the proposed system, outlining the primary components and their interconnections for a clear overview of the system’s structure and workflow. This diagram offers a comprehensive visual representation of the design, operational logic, and Arduino MCU pin mapping. The system is organized into five main subsystems: the pneumatic system, signal conditioning, signal processing, the user interface, and the power supply.

**Fig. 1:**
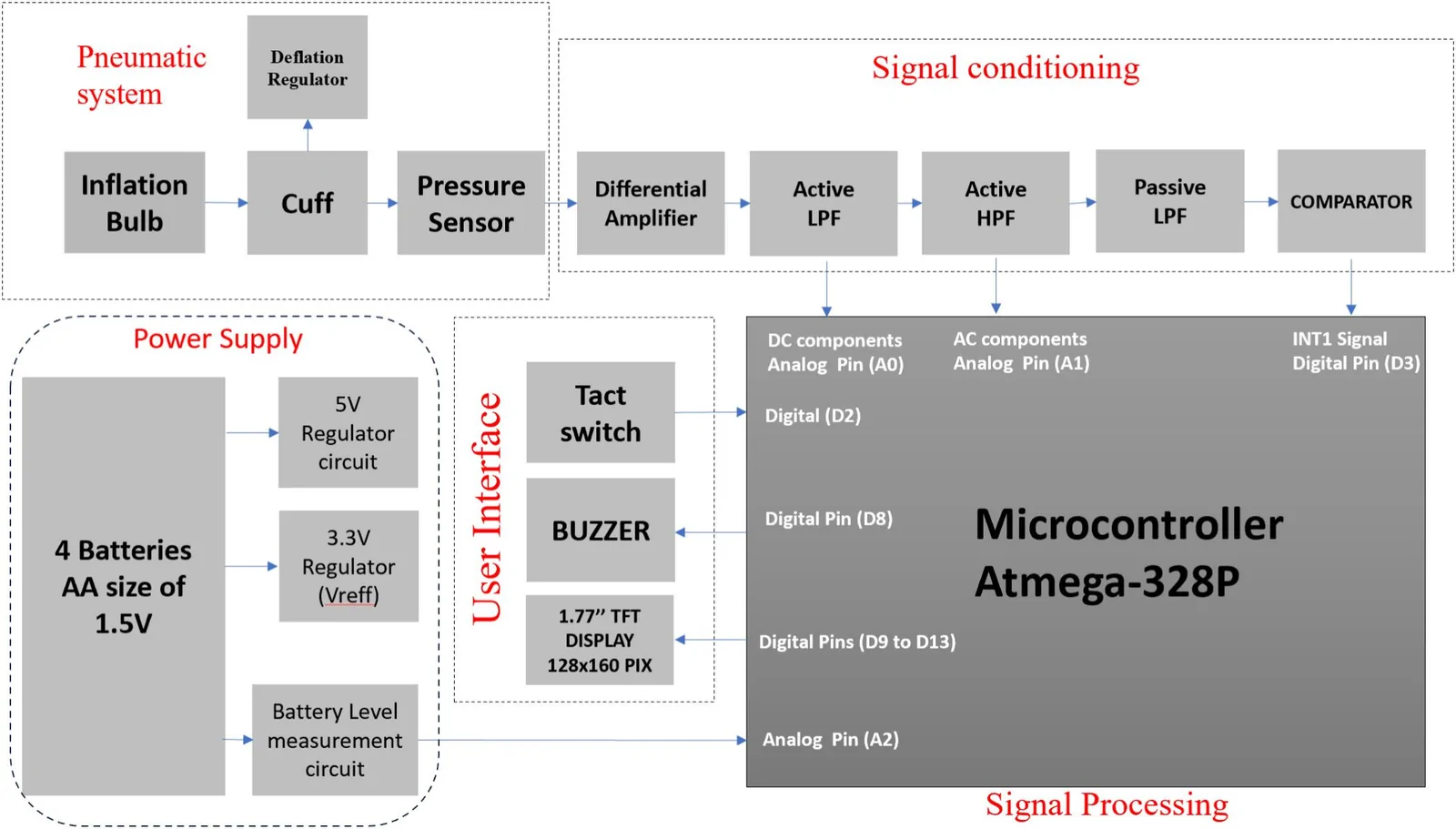
Block diagram of the proposed BP measurement device.

### 3.1 Pneumatic system design and pressure regulation mechanism

In the proposed system, the cuff is inflated manually using a hand-operated inflator, which serves as the primary pressure source for the device. A single-tube cuff system provides a pathway for inflation, with a T-connector at the end of the tube to facilitate controlled deflation, ensuring regulated airflow. Pressure release is managed through a controlled leakage (deflation) mechanism designed to release air at a constant rate, slower than the typical inflation rate, maintaining cuff pressure stability during deflation. The leakage system regulator, shown in Fig. 2, controls this release. Finally, a pressure sensor measures the cuff pressure, generating an electrical signal that is then processed and recorded for analysis.

**Fig. 2:**
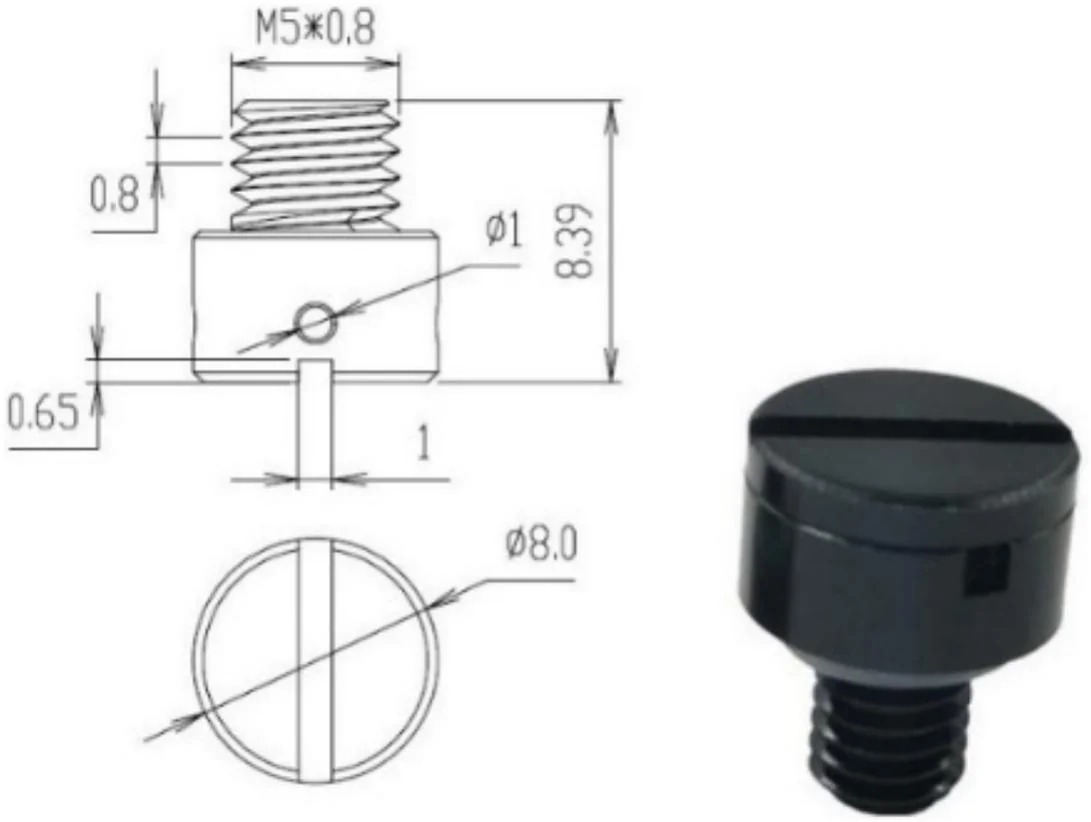
Air deflation regulator.

Since oscillometric accuracy is strongly dependent on the deflation speed, the cuff deflation rate was calibrated by monitoring the pressure-sensor output during controlled leakage. The calibration measured the time required for the pressure signal to decrease from an ADC value of 500 to 400, corresponding to a pressure drop of approximately 22.2 mmHg. The calibration was accepted when this pressure drop occurred within 9–10 s, corresponding to a deflation rate of approximately 2.22–2.47 mmHg/s. Based on repeated measurements during device operation, the overall average deflation rate was 2.53 ± 0.53 mmHg/s.

### 3.2 Pressure sensor and signal conditioning

The system uses the MPS20N0040D-S pressure sensor, which functions as a sphygmomanometer, to measure the air pressure in the cuff. It initially outputs a voltage ranging from 2.3 mV to 30 mV. A differential amplifier with a gain of 100 is employed to increase the voltage to a usable level, boosting the output to a range of 0.23 V to 3 V. This signal then passes through an active low-pass filter (LPF) with a cutoff frequency of 10 Hz and a gain of 1. This filtered signal represents the DC component of the cuff pressure and is directly sent to an MCU analog channel, specifically A0 in the Arduino pin mapping.

The DC component also takes another path, passing through an active high-pass filter (HPF) with a cutoff frequency of 2 Hz, which removes the DC component and low-frequency noise. The signal then passes through a passive low-pass filter with a 16 Hz cutoff frequency to further eliminate noise from the operational amplifier in the high-pass filter before being applied to the input of the comparator op-amp. This pathway isolates the AC component of the cuff pressure, which is then fed directly into the A1 pin of the MCU. To enhance digital signal processing speed and accuracy, a comparator converts the AC analog signal into a digital form before sending it to the digital interrupt pin (D3/INT1). The LM324 op-amp series consists of low-cost, quad operational amplifiers with true differential inputs. They have several distinct advantages over standard operational amplifier types in single-supply applications. A single op-amp chip is used to implement all active circuits: the differential amplifier, LPF, HPF, and comparator.

The device’s main power source consists of four 1.5 V AA alkaline batteries connected in series, providing 6.4 V when fresh. Since the MCU, pressure sensor, and TFT display could be damaged if exposed to 6.4 V, a Zener voltage regulator circuit is designed to supply approximately 5.1 V, as shown in the schematic diagram (Fig. 3). Additionally, a voltage regulator, the AMS1117-3.3 V, ensures a stable 3.3 V supply. The MCU’s VREF pin is connected to this 3.3 V source to maintain compatibility and enhance measurement accuracy. This reference voltage stabilizes analog-to-digital converter (ADC) readings, preserving the precision of the pressure sensor data, particularly as the battery voltage decreases over time. The battery level is measured using a voltage divider circuit to ensure that the voltage reading falls within the ADC dynamic range.

**Fig. 3:**
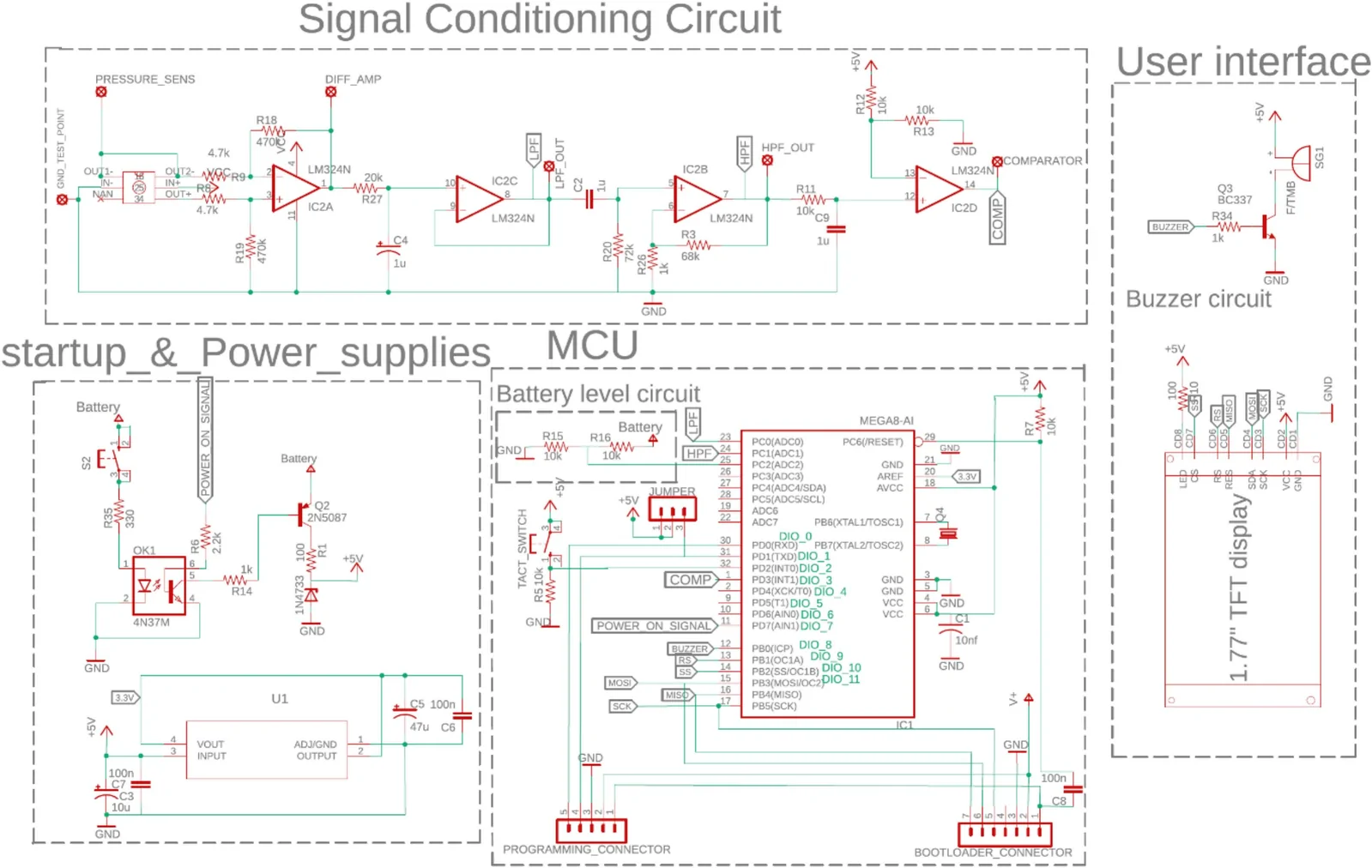
Circuit diagram of the proposed system.

An oscillometric algorithm is implemented within the MCU to analyze the discrete pressure data and calculate the heart rate (HR) and blood pressure (BP) values. This algorithm estimates diastolic and systolic pressures based on their correlation with the mean arterial pressure (MAP).

The system’s electronic circuit schematic is shown in Fig. 3. The PCB layout uses dual in-line (DIL) components to simplify assembly, enabling the easy installation of electronic components with minimal hardware requirements. The PCB layout is illustrated in Fig. 4.

**Fig. 4:**
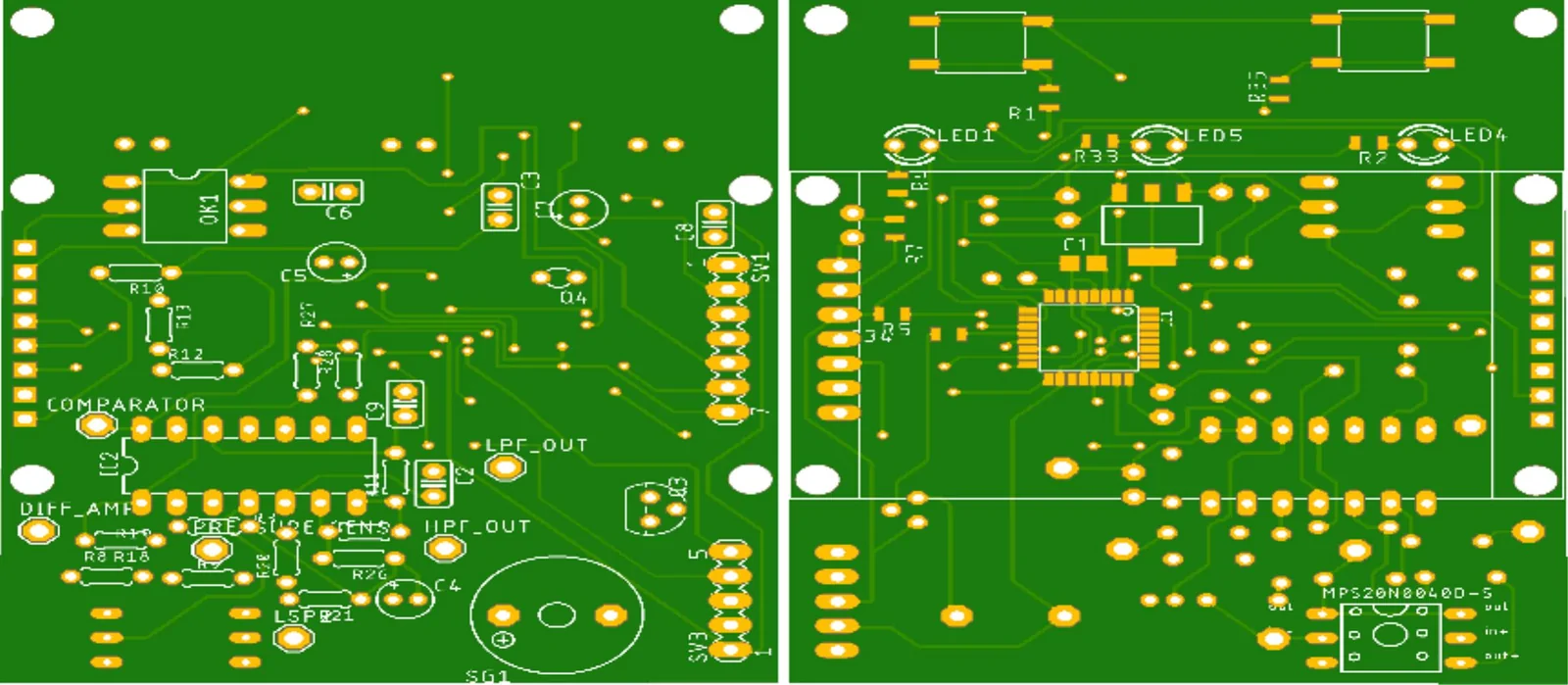
PCB layout of the proposed system.

### 3.3 Signal processing and user interface design

The blood pressure monitoring system is controlled by an ATmega328P microcontroller, which processes signals to extract pulse data and measure pressure. The chip is preloaded with the Arduino bootloader, allowing for easy programming via the Arduino Integrated Development Environment (IDE). The Bootloader_connector, shown in Fig. 3, is used for bootloader uploading. The microcontroller (MCU) separately processes the AC and DC signal components to compute key blood pressure metrics. An interrupt signal (INT) is connected to the MCU to enhance synchronization, improve accuracy by reducing processing time, and aid in detecting and eliminating artifact pulses. The system is powered by four AA batteries and features a buzzer, LEDs, and a 1.77-inch TFT display for real-time feedback. User interaction is facilitated by two tact switches: one for powering on the system, and the other reserved for future developments, such as software upgrades or additional functionality. A “JUMPER” connector can be used to connect the D1 pin to logic 1, enabling the processor to enter calibration mode, which will be described in Section 5.

After the vital signs are computed, they are displayed on a 1.77-inch SPI TFT display (128 × 160 pixels) for user observation. A buzzer, connected to the MCU’s D8 digital pin, provides audible signals to guide the user: indicating when to stop the inflation phase, beeping with each heartbeat pulse during measurement, and signaling the end of the measurement phase. To conserve battery power, a 4 N35 optocoupler is connected to the D7 digital pin of the MCU. It automatically deactivates the device during inactivity, thus ensuring efficient power management and extending battery life.

To substantiate the low power consumption claims, empirical current measurements were recorded. The device is powered by a 6 V supply (four 1.5 V AA batteries). During active measurement cycles, with the ATmega328P microcontroller, the LM324 quad op-amp, the pressure sensor, and the TFT display fully operational, the system draws a total current of approximately 50 mA, corresponding to a maximum power consumption of approximately 300 mW. When evaluating the core signal-processing and pneumatic-sensing electronics without the TFT display, the current draw is approximately 25 mA (150 mW). Assuming a nominal capacity of 2000 mAh for the AA alkaline batteries, the measured active current corresponds to an estimated continuous operating time of approximately 40 h, or approximately 1600 measurements assuming an average measurement duration of 90 s.

When the timeout period elapses or the measurement concludes, the MCU deactivates the 4 N35 optocoupler, which in turn switches off the main PNP transistor (Q1). This completely disconnects the power supply from the entire electronic circuit. Consequently, the idle/standby power consumption is reduced to the mere leakage current of the transistor (typically < 1 µA), ensuring zero effective battery drain during inactivity until the user manually presses the tact switch to initiate a new cycle. This ultra-low power architecture represents a significant advantage for prolonged deployment in resource-limited settings.

### 3.4 Printed 3D components and system assembly

In recent years, 3D printing technology has transformed from a prototyping tool into a viable solution for creating end-use products. With appropriate design and materials, reliable and robust parts can be produced by 3D printers, reducing the need for additional machinery [14]. As 3D printer prices continue to decrease globally, this technology is being widely adopted by institutions, including universities, in laboratories and research and development (R&D) departments [15]. This section introduces the main components of the system enclosure or housing.

#### 3.4.1 Printed 3D components and ready-made components

For the design of the blood pressure monitoring device, SolidWorks software was used to create and optimize the components. SolidWorks provided precise control over complex geometries, allowing specific design requirements to be met. A modular approach was taken, with the structure divided into smaller components to simplify assembly, customization, and future modifications.

No motors are used in this blood pressure device, which simplifies construction and reduces potential mechanical failure points. ABS plastic was chosen for the 3D-printed parts, including the body, cover, and buttons, due to its durability and resistance to high temperatures. The 3D-printed components and their design details are illustrated in Fig. 5. This material selection was essential to ensure the device’s longevity under prolonged use without deformation. This modular design approach allows for flexibility in modifying individual parts while optimizing dimensions and structural integrity. By using SolidWorks and a 3D printer, the blood pressure device was prototyped and refined quickly, with each component verified to meet functional and ergonomic requirements. This integration of CAD software and 3D printing has resulted in a cost-effective, reliable, and customizable device suitable for practical healthcare applications. Furthermore, Fig. 6 illustrates the ready-made components, highlighting their dimensions and real-life appearance.

**Fig. 5:**
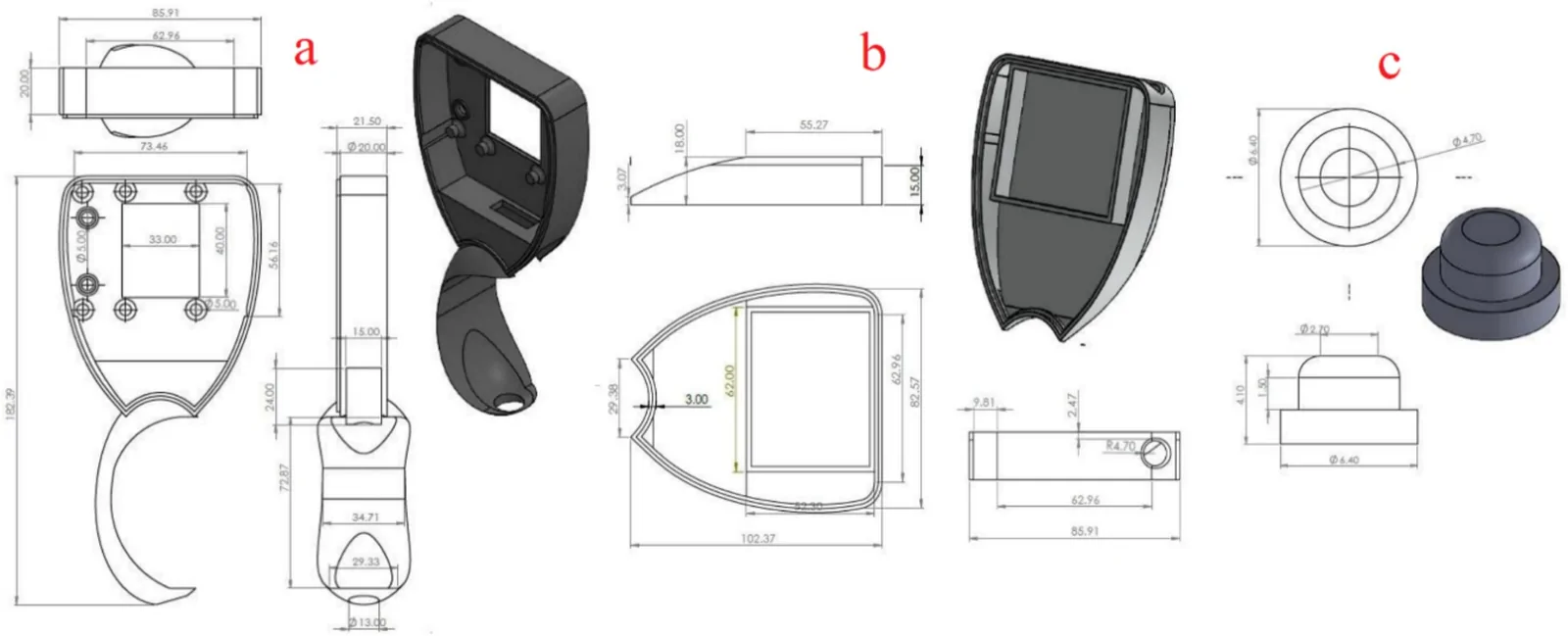
Printed 3D CAD design for; (a) secure cover, (b) device grip and frame, and (c) tact switch Cap design.

**Fig. 6:**
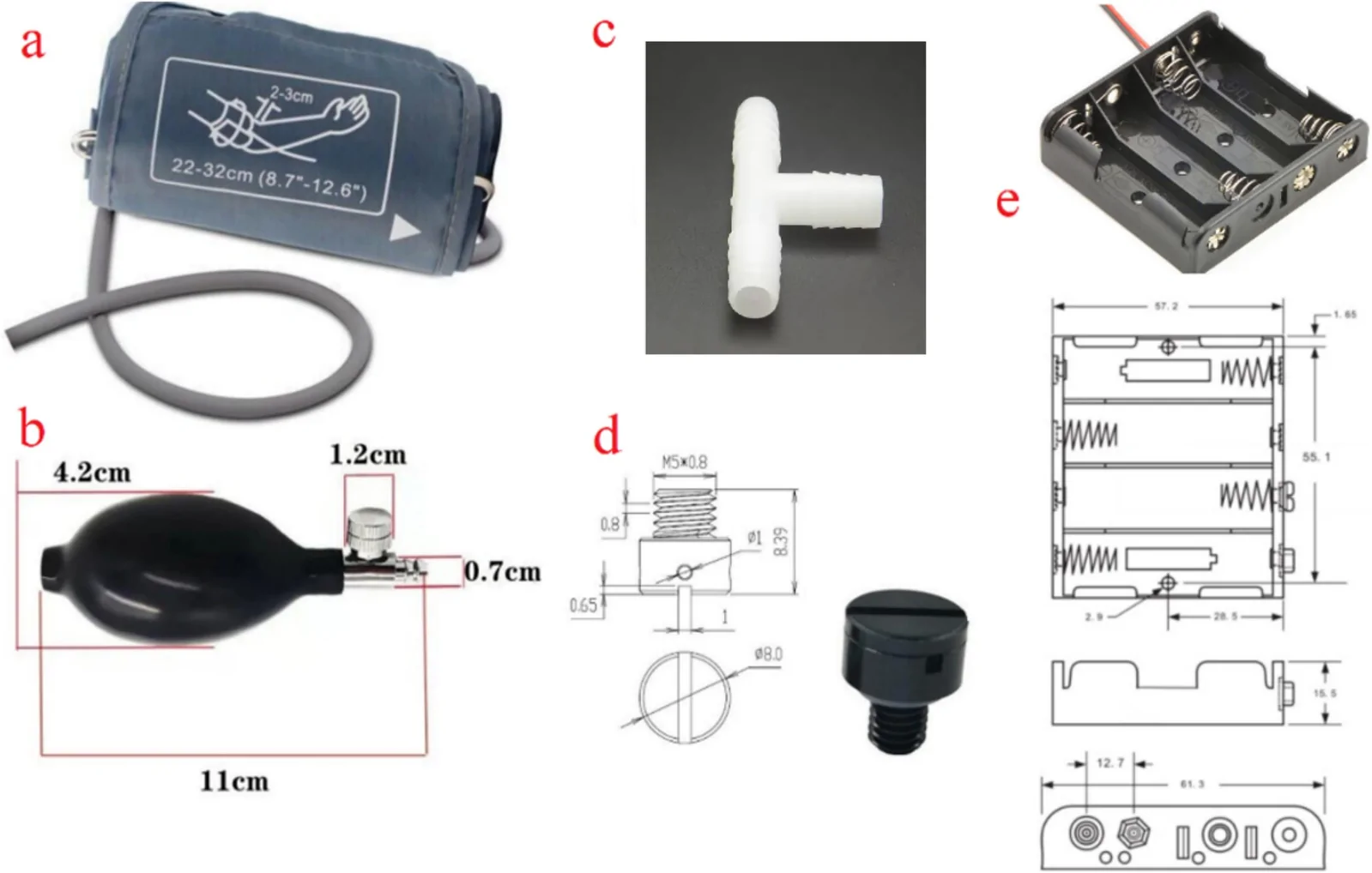
ready-made components; (a) arm cuff, (b) inflation bulb, (c) arm cuff T-connector, (d) air deflation regulator, and (e) battery power bay.

#### 3.4.2 System assembly

Fig. 7 illustrates the integration of the 3D-printed parts with the other components of the device. The fully assembled device is shown on the left side of the figure, while the individual components are displayed on the right side. The design incorporates a streamlined assembly process, ensuring a compact and functional device.

**Fig. 7:**
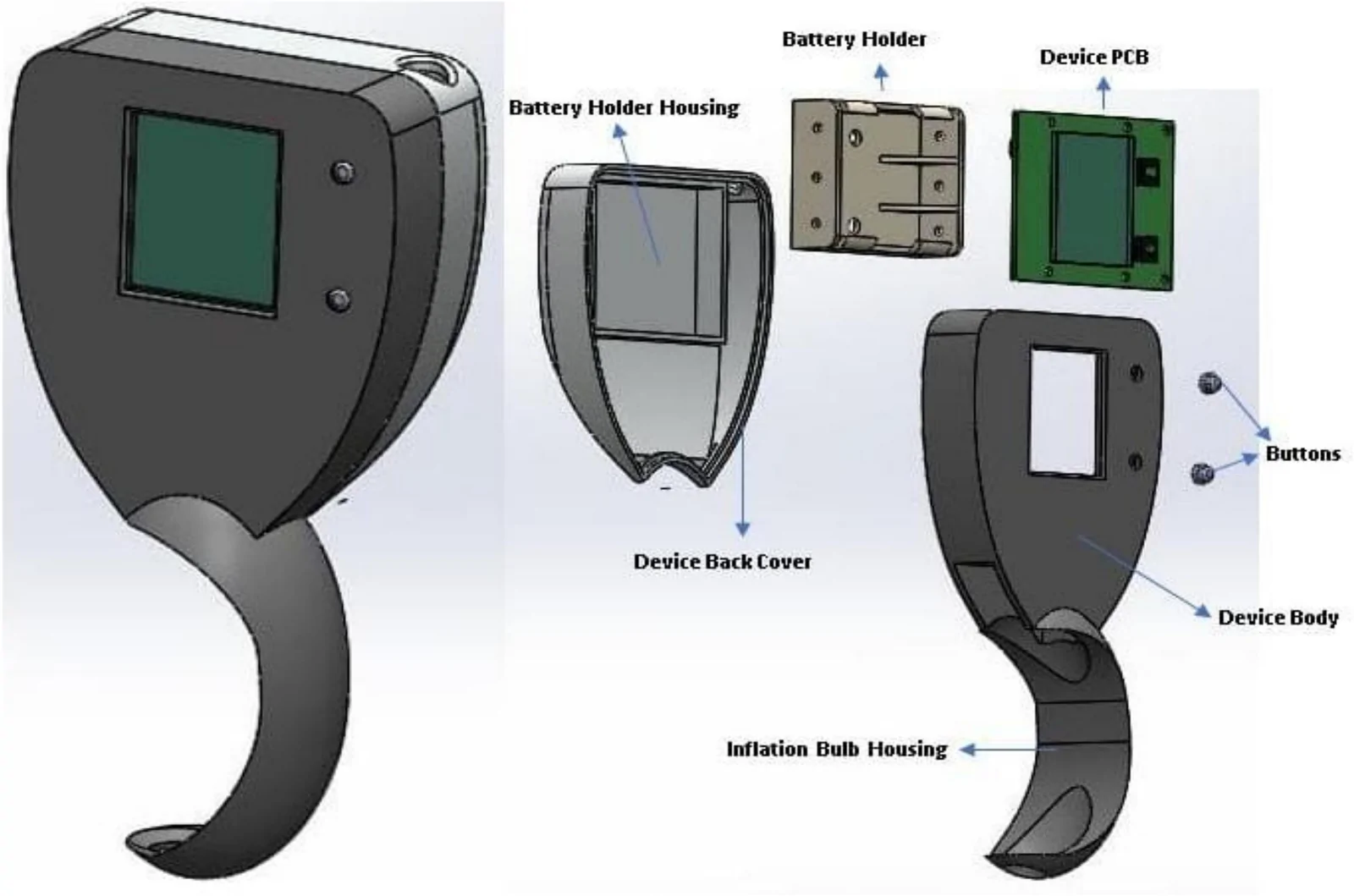
Complete Assembly of the Blood Pressure Device.

The device consists of several key components that work together seamlessly. The main structural part, referred to as the device body, houses the screen, buttons, and the ergonomic handle. Inside the body, the device’s PCB is mounted to provide electronic functionality. Additionally, a battery holder and its corresponding housing are designed to securely accommodate the batteries and establish a reliable electrical connection. To enclose the internal components, a detachable back cover is included, allowing for easy access to the battery compartment for maintenance or replacement.

On the left side of the figure, the assembled device showcases its key features, including a clear screen for data display and two push-button switches for user interaction. The upper switch is designated for powering on the device, while the lower switch serves as an idle switch reserved for future functionalities.

The curved handle at the bottom of the device is designed to securely hold the inflation bulb, as shown in Fig. 6(b). This ergonomic feature ensures ease of handling and stability during operation, contributing to the device’s overall user-friendly design.

### 3.5 Software design and algorithm development

The Arduino IDE software was employed to manage the processing of pressure data and to control the display. The developed algorithm, which interacts with interrupt signals and employs artifact reduction techniques, is one of the developed strategies implemented in this proposed blood pressure measurement device. The algorithm, depicted in the flowchart in Fig. 8, is divided into three subroutines: the main loop, positive-edge interrupt, and negative-edge interrupt.

**Fig. 8:**
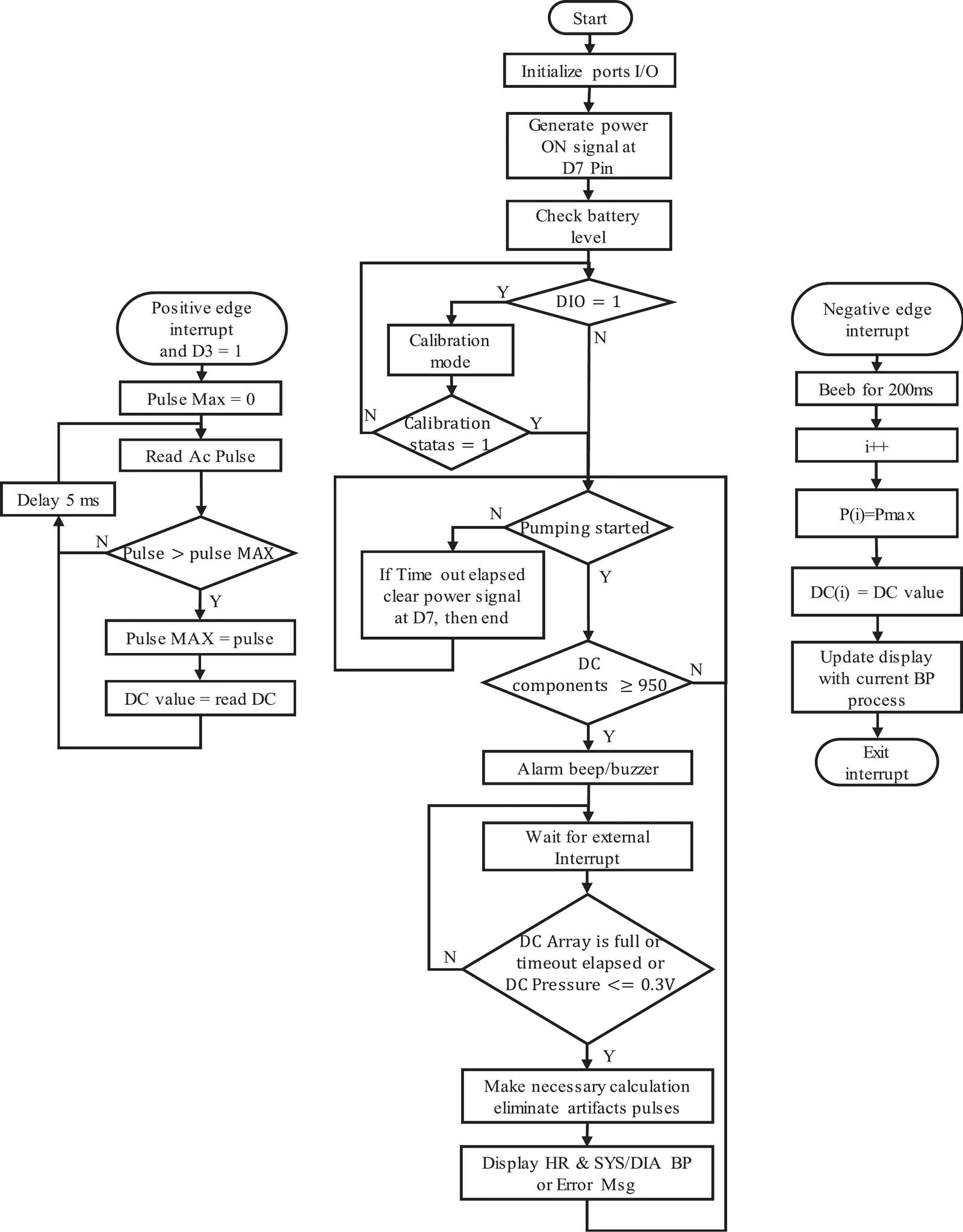
Software flowchart.

When the start button is pressed, an optocoupler activates its internal transistor, which, in turn, switches on the PNP transistor Q1. This action connects the power supply to the electronic circuit, thereby initiating the system. The system then initializes by setting up the required components, including input/output ports, interrupts, and analog channels for the pressure sensor. This preparation ensures that the microcontroller can handle both positive**-** and negative-edge interrupts as needed during operation. During initialization, the system is configured to monitor AC pulses from the pressure sensor and measure DC voltage to determine pressure levels. Once initialization is complete, the microcontroller sends a logic “1″ signal to the base of the transistor (connected to the Arduino D7 pin), maintaining the system in an ON state after the switch is released. If, within 20 s, the pressure sensor does not exceed 3 V (equal to 200 mmHg), the system deactivates by sending a logic ”0″ to the transistor to power down and save battery energy. The system also has the option to enter calibration mode, which will be illustrated in Section 5.

After initialization, the main loop starts, where the system continuously monitors the inflation process, handles interrupts, and processes sensor data. During operation, the microcontroller tracks the DC pressure levels while the inflation is running. If the pressure reaches or surpasses 3 V, an alarm sounds to signal a critical threshold, and the user should stop inflation. The system then enters a waiting state, monitoring for an external interrupt, a timeout, or a pressure drop below 0.5 V, corresponding to approximately 30 mmHg based on the calibrated pressure-sensor relationship implemented in the device software. If the timeout period (10 s) expires without any interrupt phase, an error message is displayed, and the system shuts off. When the pressure drops below the threshold or 50 pulses are detected (reaching the array size), the measurement process completes. Artifact pulses are then eliminated based on the average time calculation between pulses. Next, the systolic, diastolic, and heart rate values are calculated using data acquired from positive**-** and negative-edge interrupts and then displayed. The system then waits for external input to proceed or shuts down after a period of inactivity.

The positive**-**edge interrupt subroutine primarily detects the peak value of each pulse. When a positive-edge interrupt occurs, the loop will keep searching for the maximum value while the digital value at the D3 pin is “HIGH”. Then, the system initiates pulse data measurement, resetting the pulse max value to zero and reading the current AC pulse from the pressure sensor. If this pulse sample value is higher than the previous maximum, the pulse max value is updated. The corresponding AC and DC voltages are recorded in two identically sized arrays, which support the oscillometric data required to calculate blood pressure values.

In a negative**-**edge interrupt, which signals the end of the AC pulse, the processor is free as no new maximum pulse value needs to be tracked. During this interval, the system generates a beep for 200 ms and updates the display with the current cuff pressure readings. The display shows the instantaneous pressure reading before returning to the main loop. The stored DC and pulse values are then processed to calculate precise BP readings for display, ensuring real-time feedback during BP measurement.

The main loop continues this cycle of monitoring, handling interrupts, and updating until the deflation completes its operation. The entire algorithm relies on precise timing, data collection, and interrupt-driven processes to ensure accurate and real-time BP measurements. By combining alarm signaling, pulse detection, and display updates, the system provides an efficient flow from initiation through completion of the BP measurement process.

Unlike auscultatory measurement, oscillometric SBP and DBP estimation is empirical. There is no universal physiological ratio that defines systolic and diastolic pressures. During cuff deflation, the oscillometric pulse amplitudes increase to a maximum at the Mean Arterial Pressure (MAP) and then decrease. SBP is determined when the oscillation amplitude reaches a percentage of the maximum on the rising side, while DBP is determined when the oscillation amplitude reaches a percentage of the maximum on the falling side, as seen in Fig. 20.

Previous studies have reported different systolic and diastolic detection ratios for oscillometric BP estimation. Geddes et al. reported SBP and DBP ratios of 0.45–0.57 and 0.75–0.86, respectively, in human subjects, while broader ranges of 0.45–0.73 for SBP and 0.69–0.83 for DBP have also been reported [16], [17], [18]. In the present study, multiple systolic and diastolic ratios were evaluated using the collected validation dataset, and ratios of 0.47 for SBP and 0.78 for DBP produced the lowest mean absolute error relative to the reference measurements and were therefore adopted. These values are consistent with previously reported ratios and demonstrate that the optimal coefficients are dependent on the device and signal-processing approach.

### 3.6 Potential applications

• Low-cost blood pressure measurement: The device can be used as an affordable platform for BP measurement in laboratory and educational environments.
• Medical engineering education: The open-source hardware provides a practical platform for students and researchers to study pressure sensing, signal conditioning, oscillometric BP estimation, and embedded-system implementation.
• Algorithm development and testing: Researchers can use the accessible hardware and acquired pressure signals to develop and evaluate alternative pulse-detection, artifact-rejection, and BP-estimation algorithms.
• Hardware and system development: The modular design can serve as a starting point for researchers developing or testing modifications to BP monitoring systems, including alternative sensors, displays, signal-processing methods, or user interfaces.

## 4 Design files summary

Table 1.

**Table 1:** Design files summary.

Design file name	File type	Open source license	Location of the file
Device top-frame and Grid	SolidWorks CAD file (.SLDPRT)	MIT License	**10.17605/OSF.IO/4RAB3**
Device top-frame and Grid	3D_printing file (.stl)	MIT License	**10.17605/OSF.IO/4RAB3**
Device bottom frame (Cover)	SolidWorks CAD file (.SLDPRT)	MIT License	**10.17605/OSF.IO/4RAB3**
Device bottom frame (Cover)	3D_printing file (.stl)	MIT License	**10.17605/OSF.IO/4RAB3**
Tact Switch Cap	SolidWorks CAD file (.SLDPRT)	MIT License	**10.17605/OSF.IO/4RAB3**
Tact Switch Cap	3D_printing file (.stl)	MIT License	**10.17605/OSF.IO/4RAB3**
4-ways Air Pipes connector	SolidWorks CAD file (.SLDPRT)	MIT License	**10.17605/OSF.IO/4RAB3**
4-ways Air Pipes connector	3D_printing file (.stl)	MIT License	**10.17605/OSF.IO/4RAB3**
*PCB schematic*	Eagle CAD design (.sch)	MIT License	**10.17605/OSF.IO/4RAB3**
*PCB layout*	Eagle CAD design (.brd)	MIT License	**10.17605/OSF.IO/4RAB3**
*PCB gerbers*	Compressed folder (.Zip)	MIT License	**10.17605/OSF.IO/4RAB3**
Arduino_Code	Arduino (.ino)	MIT License	**10.17605/OSF.IO/4RAB3**
Valve_Calibration Code	Arduino (.ino)	MIT License	**10.17605/OSF.IO/4RAB3**

## 5 Bill of materials summary

.

**Table 2:** Bill of Materials (BOM).

Designator	Component	Number	Cost per unit −currency	Total cost − currency	Source of materials	Material type
Inflation bulb (Fig. 6(b))	Black Manual Air Pump Inflation Sphygmomanometer Oximetro Mercury Blood Pressure Latex Ball Bulb + Air Release	*1*	0.47	0.47	Bulb_Link	*Select from:* − Metal − High Quality Natural Latex rubber
Cuff (Fig. 6(a))	22–48 cm Adult Blood Pressure Cuff for Arm Blood Pressure Monitor Meter Tonometer Sleeves Sphygmomanometer Belt	1	1.33	1.33	Cuff_link	− Polyester − PVC
Atmega-328P	SMD TQFP32 chip microcontroller IC chip	1	1.17	1.17	MCU_Link	Semiconductor
LM324	DIP-14 Operational Amplifiers Op Amps Quad Operational Amp	1	0.064	0.064	Op-Amp_Link	Semiconductor
TFT 1.77″ screen	TZT 1.77 in. TFT LCD Screen 128160_SPI, TFT Color Screen Module Serial Port Module For Arduino UNO R3	1	1.55	1.55	TFT_Link	Semiconductor
Electronic components	Resistors, capacitor…etc	Lumped sum	1.0	1.0	N/A	− Semiconductor − Carbon − others
3D printing	ABS 150 g	1	2	2	N/A	ABS
Battery Socket (Fig. 6(e))	JCD 4 Slot AA Battery Case Box, AA Battery Holder	1	0.2	0.2	Socket_Link	− Polycarbonate
Release valve (Fig. 6(d))	Adjustable Air Permeable IP67 Metal M5 Breather Vent Plug for Portable Electronics	1	0.21	0.21	Valve_link	N/A
PCB (Fig. 4)	1.6 mm thinckness PCB	1	0.4	0.4	www.JLCPCB.com	FR-4

## 6 Build instructions

This section describes the process of constructing the proposed PressSure blood pressure device. The construction is carried out in three stages: assembling the circuit on a PCB, uploading the software, and completing the final assembly by attaching the 3D-printed plastic cover.

### 6.1 PCB assembly

Begin by assembling the printed circuit board (PCB), carefully soldering all electronic components in accordance with the schematic provided in Fig. 3. The assembly process should follow a logical sequence: start by mounting and soldering the ATmega328P microcontroller, as illustrated in Fig. 9b. Next, proceed with the surface-mount devices (SMDs), followed by the through-hole (THT) components, which can be referenced in Fig. 9c. This sequence helps reduce handling stress and ensures unobstructed access during each step of the process. The design employs dual in-line (DIL) components where applicable to simplify manual soldering and facilitate future replication. For small SMDs, such as those in 0805 or 0603 packages, a hot air rework station is advised to achieve precision and reliability. Although stencil-based solder paste application enhances uniformity, manual soldering was preferred during the prototyping phase to allow for greater flexibility and iterative testing.

**Fig. 9:**
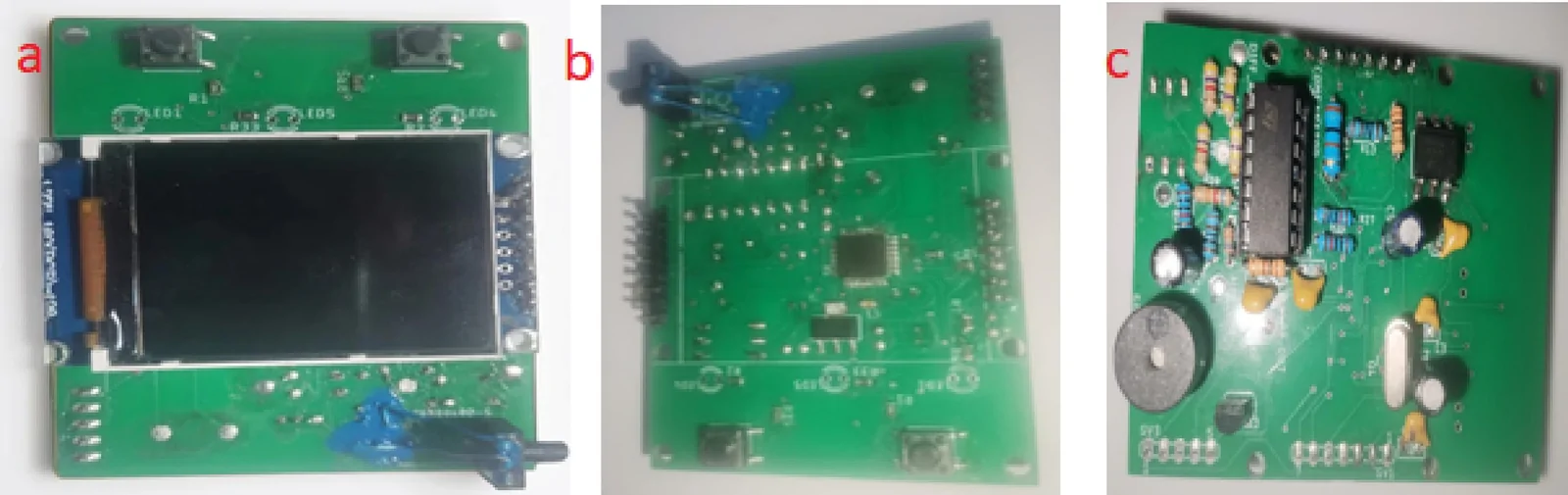
PCB assembly for; (a) TFT display, (b) ATmega328P microcontroller, and (c) THT components.

Beyond passive components and integrated circuits, the 1.77-inch SPI TFT display (128 × 160 pixels) must be soldered directly onto the PCB using a suitable header pin connector. Careful attention should be paid to its alignment and connection quality, as this display serves as the main visual interface for presenting real-time blood pressure and heart rate data.

After completing all soldering and performing a thorough visual inspection, the assembled PCB, including the mounted TFT display, can be seen in Fig. 9a. At this stage, it is advisable to verify electrical continuity and module functionality before moving on to mechanical integration.

### 6.2 Burning bootloader to ATmega328P using Arduino Uno R3

Burning the bootloader onto an ATmega328P microcontroller using an Arduino Uno R3 is a crucial step in preparing a blank microcontroller for Arduino-based applications. The bootloader enables the ATmega328P to communicate with the Arduino IDE via a serial connection, allowing future code uploads through a USB-to-serial interface. In this setup, the Arduino Uno functions as an in-system programmer (ISP), transferring the bootloader to the target ATmega328P, which is typically mounted on a custom PCB along with essential supporting components such as capacitors, resistors, and a crystal oscillator.

To initiate the process, upload the “ArduinoISP” sketch to the Uno via the Arduino IDE, as illustrated in Fig. 10a. Next, establish the required connections between the Uno and the ATmega328P: pin 10 (RESET), pin 11 (MOSI), pin 12 (MISO), pin 13 (SCK), along with GND and 5 V. These connections allow the Uno to directly interface with the ATmega328P and burn the bootloader, as shown in Fig. 10c.

**Fig. 10:**
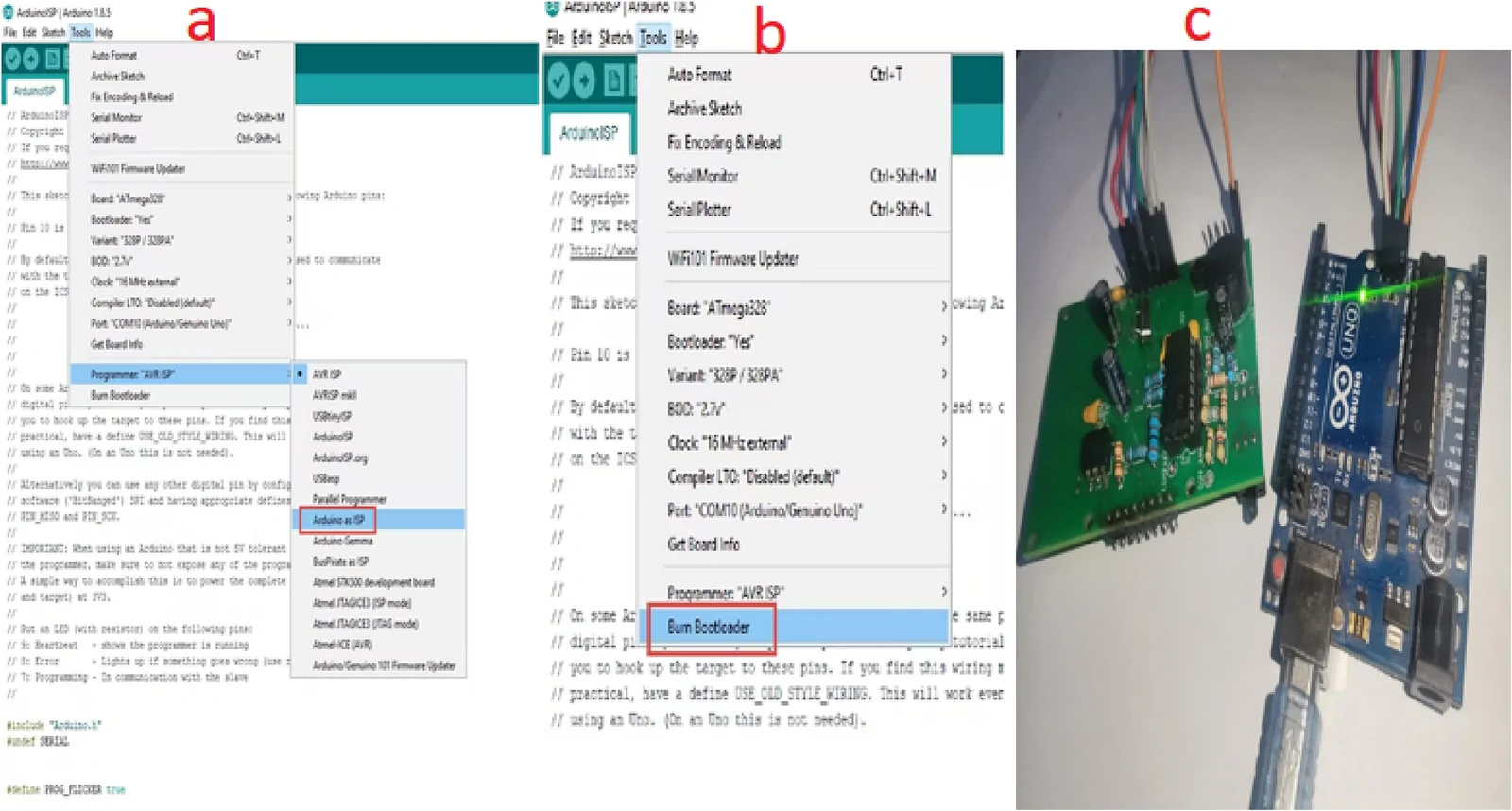
Bootloader burning process; (a) ArduinoISP Sketch, (b) Bootloader burning command, and (c) Arduino Uno and the ATmega328P connection.

It is strongly recommended to perform this bootloader burning procedure before soldering the 1.77-inch TFT screen to the PCB. Doing so ensures unobstructed access to the ISP pins, which may become difficult to reach once the display is mounted. With the wiring in place, select “Arduino as ISP” from the “Programmer” menu in the Arduino IDE and ensure the correct board and COM port are selected. Clicking “Burn Bootloader,” as depicted in Fig. 10b, initiates the process and programs the ATmega328P, enabling it to receive sketches via USB.

This step is especially important for developers working with embedded systems and custom hardware, as it prepares the microcontroller for reliable and efficient prototyping, while avoiding potential complications caused by permanently mounted components like the TFT display during early development.

### 6.3 Uploading code via USB to TTL to standalone ATmega328P

Uploading software to an ATmega328P microcontroller using an FTDI FT232R USB-to-TTL serial adapter is a widely adopted and efficient method for programming standalone embedded systems. The FTDI module serves as a bridge between the computer’s USB port and the microcontroller’s UART interface, enabling seamless communication with the Arduino IDE. In the configuration illustrated in Fig. 11b, the FTDI adapter is connected as follows: TX to the ATmega328P’s RX (pin 2), RX to TX (pin 3), GND to GND, VCC to VCC (5 V), and DTR to the RESET pin. Notably, a 0.1**-**µF capacitor is already integrated on the PCB between the DTR line and the RESET pin. This capacitor is essential, as it allows the microcontroller to automatically enter bootloader mode during uploads, eliminating the need for manual resets. Its role is to ensure that a correctly timed reset signal is delivered when uploading code.

**Fig. 11:**
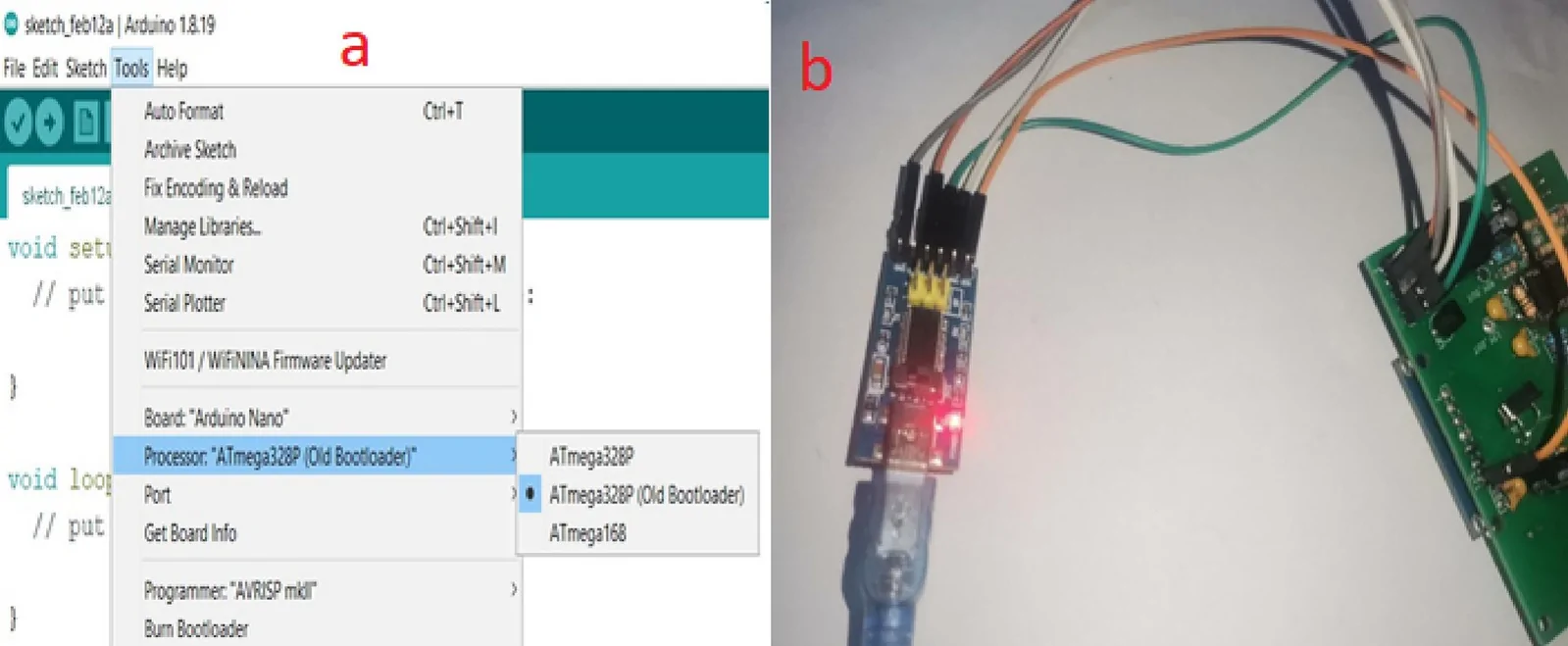
Code Uploading process; (a) Board and processor selection, and (b) ATmega328P wit USB to TTL connection.

Within the Arduino IDE, it is important to select the appropriate board and processor settings that match the bootloader version programmed onto the ATmega328P. In this scenario, users should select “Arduino Nano” as the board and “ATmega328P (Old Bootloader)” as the processor, as shown in Fig. 11a. This is because many standalone ATmega328P chips come preloaded with the older bootloader, which uses different upload timing from the newer version. After selecting the correct COM port, the sketch can be uploaded directly via the FTDI adapter. This programming method offers a reliable and convenient solution for developers and researchers working with compact, USB-less embedded systems. It enables efficient code updates and rapid prototyping on custom-designed PCBs without the complexity of additional hardware components.

### 6.4 Pneumatic component integration

The pneumatic integration begins by connecting a short silicone tube to the pressure port of the MPS20N0040D-S pressure sensor, as shown in Fig. 12a. This tube acts as the channel for transmitting the air pressure from the cuff, depicted in Fig. 12b, to the sensor. Through this connection, the applied pressure is accurately conveyed, allowing the sensor to capture reliable signals for subsequent measurement and analysis.

**Fig. 12:**
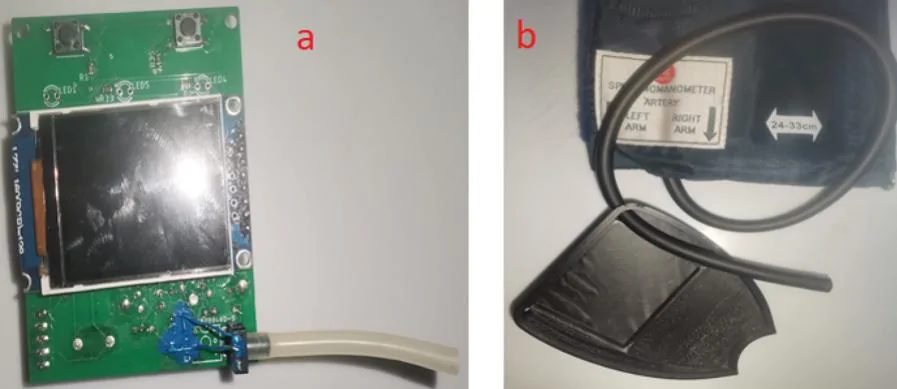
(a) pressure sensor tube connection, and (b) cuff tube preparing.

Next, the manual inflation bulb is prepared by attaching a short silicone tube to its outlet, as shown in Fig. 13a. This bulb serves as the primary means of manually inflating the cuff by pumping air into the system. In parallel, another short silicone tube is connected to the air deflation regulator, as illustrated in Fig. 13b. The regulator enables the controlled release of air pressure from the cuff, allowing for gradual deflation during measurement and ensuring accurate oscillometric signal detection.

**Fig. 13:**
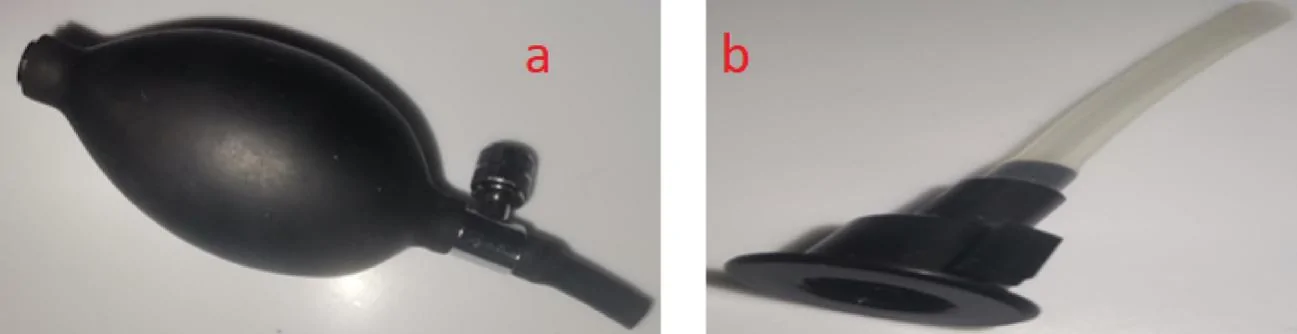
(a) bulb tube connection, and (b) regulator tube connection.

### 6.5 Mechanical integration with the 3D-printed body

All 3D-printed components, including the body, cover, and button cap, were produced using ABS + filament. This material, printed with 100% infill and a 0.2 mm layer height, provides the strength, durability, and thermal resistance necessary for the device. The design of these components was meticulously refined using SolidWorks, resulting in a high-quality and functional structure, as illustrated in Fig. 5.

The mechanical integration of the 3D-printed enclosure begins with mounting the tact buttons into their designated positions on the device’s structure, as shown in Fig. 14a. Next, the assembled printed circuit board (PCB) is carefully positioned inside the housing and securely fastened using screws to ensure both stability and proper alignment, as illustrated in Fig. 14b. Finally, the cuff’s main air tube is routed through the top opening of the enclosure, allowing for seamless pneumatic connectivity with the internal components.

**Fig. 14:**
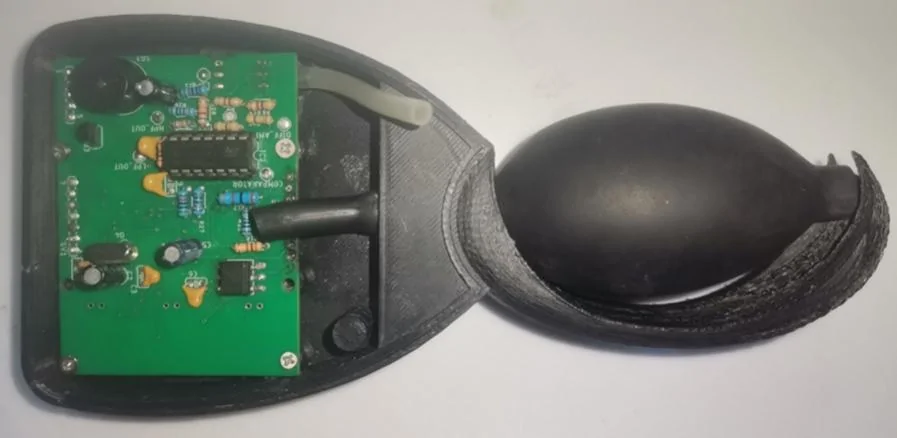
(a) tact buttons placing, and (b) PCB placing.

Next, the inflation bulb is inserted into an ergonomic holder integrated into the bottom grip of the device, as shown in Fig. 15. This design ensures user comfort during operation while maintaining the secure placement of the bulb. These steps collectively guarantee that all essential mechanical components are accurately positioned and firmly secured within the device’s framework, thereby supporting optimal performance and ease of use.

**Fig. 15:**
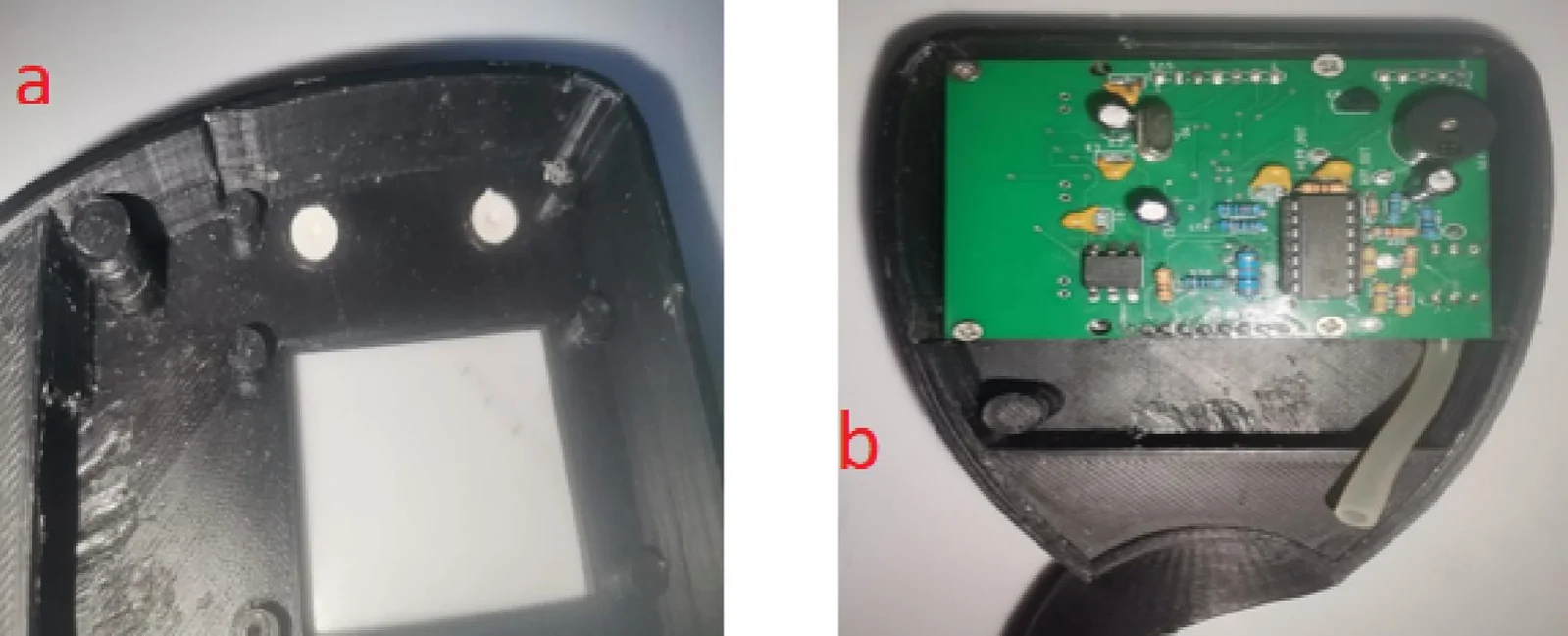
bulb preparing.

### 6.6 Pneumatic system assembly and configuration

To consolidate the pneumatic system, a four-way T-connector is used as the central hub for air distribution. As shown in Fig. 16a, the air deflation regulator is connected to one port of the T-connector via a short silicone tube. Another port is linked to the pressure sensor using a similar silicone tube, as illustrated in Fig. 16b. The manual inflation bulb is directly connected to a third port of the T-connector, as shown in Fig. 16c. The fourth port interfaces with the cuff through an additional silicone tube, completing the pneumatic loop. This configuration allows for simultaneous inflation, controlled deflation, and real-time pressure sensing across all components.

**Fig. 16:**
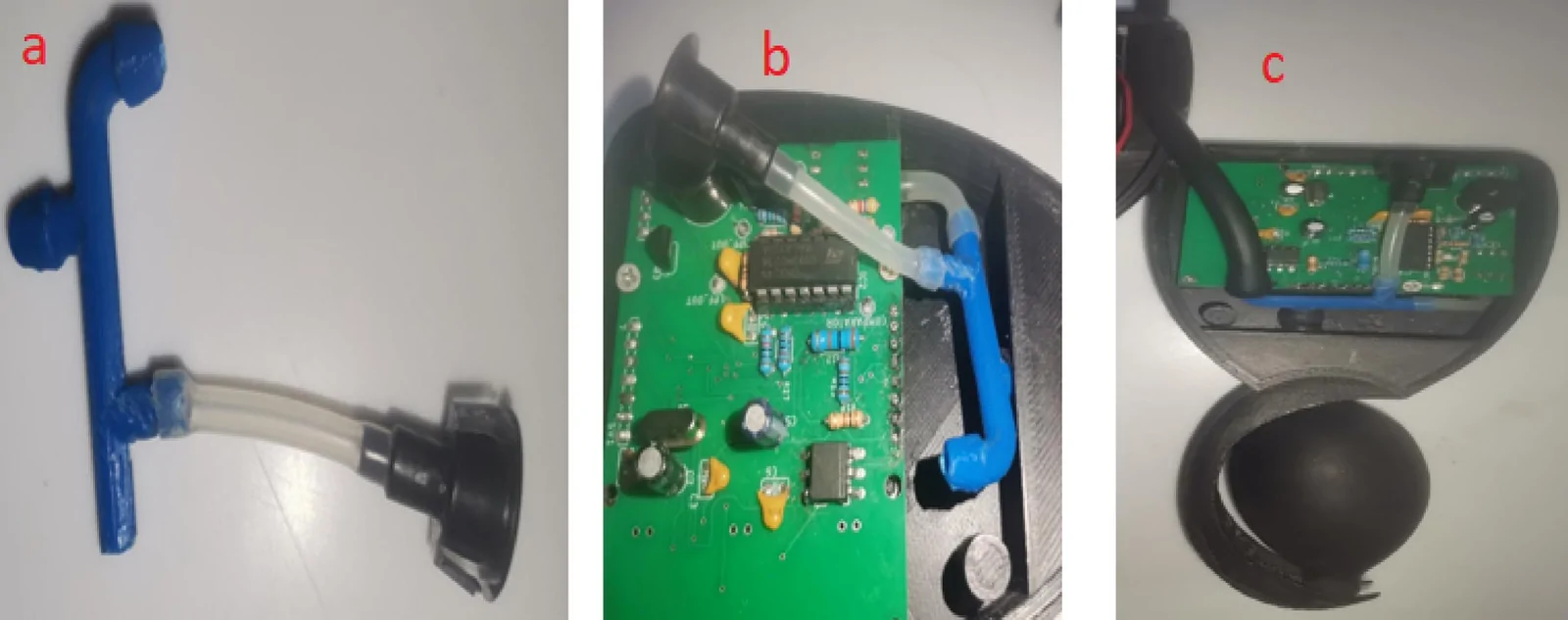
shows the connection for; (a) regulator and T-con., (b) pressure sensor and T-con., and (c) bulb and T-con.

To ensure accurate pressure transmission and reliable operation, each pneumatic connection must be tightly secured to maintain an airtight seal. A small amount of adhesive is recommended at each joint to prevent potential air leaks and enhance the long-term stability of the tubing connections.

### 6.7 Power supply and final assembly

The power supply setup begins by mounting the four-slot AA battery holder onto the inner side of the device’s back cover, as depicted in Fig. 17a. The holder can be secured in place using a suitable adhesive. Ensuring a stable attachment is crucial for the reliable functioning of the power source within the device.

**Fig. 17:**
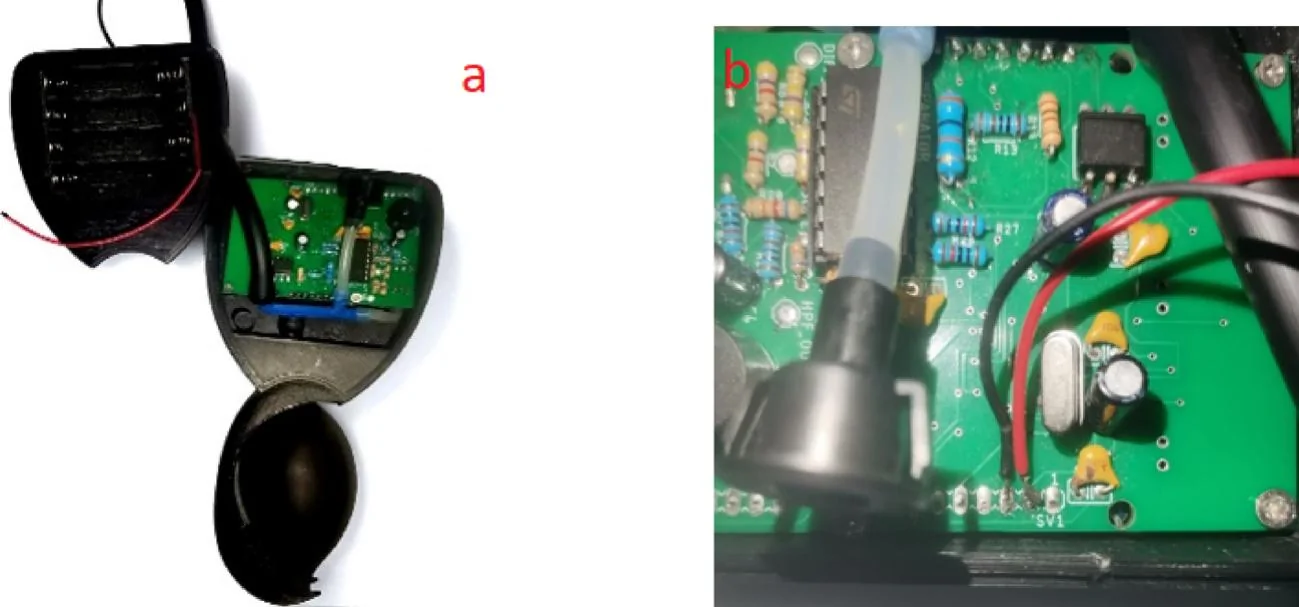
(a) four-slot AA case pleasing, and (b) power connection in PCB.

After installing the battery holder, the wires are carefully connected to the power terminals on the printed circuit board (PCB), ensuring correct polarity, as shown in Fig. 17b. Once all electrical connections are made, a thorough verification process is conducted to check both the electrical and pneumatic systems, ensuring proper integration and operational readiness.

Finally, the assembly process is completed by aligning the top cover with the body of the device and fastening them securely to form the enclosure. At this point, the device is fully assembled, as shown in Fig. 18. It is now prepared for functional testing, calibration, and eventual operation, as outlined in Section 7.

**Fig. 18:**
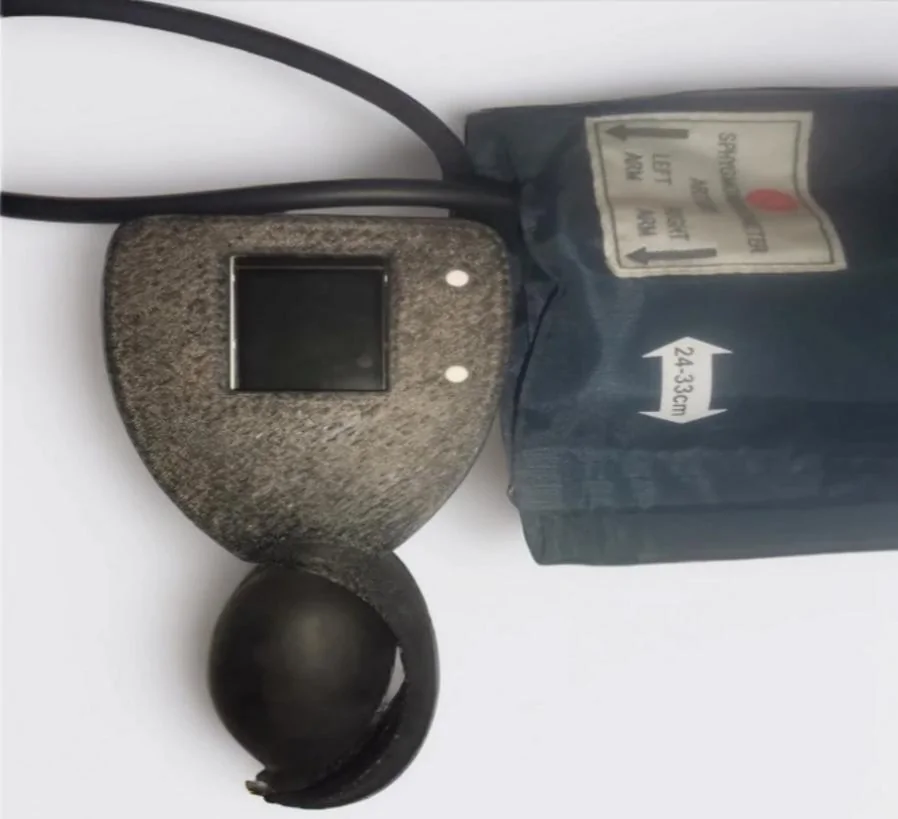
fully assembly blood pressure device.

## 7 Operation instructions

Once calibration is performed by the developers or engineers, the proposed blood pressure device offers highly user-friendly operation. The system operates via a single push button, ensuring simplicity for users. Upon activation, the device provides clear on-screen messages at each stage, as outlined in the algorithm in Section 4. The user must manually inflate the cuff using the bulb and reach the required pressure threshold within 20 s. If this time limit is exceeded, the system automatically shuts down. Additionally, if the pressure surpasses the predefined safe limit, a long beep from the buzzer alerts the user to stop pumping. After reaching the target pressure, the user must stop pumping and wait for the measurement process to complete, which typically takes 45 to 55 s after the beep. Upon completion, the screen displays the heart rate (HR), diastolic pressure, and systolic pressure. The system then remains active for 15 s before resetting to its initial state, awaiting a new inflation cycle. If no inflation occurs within the following 20 s, the device automatically powers off to conserve battery life.

### 7.1 Step-by-step measurement process

a. Preparation:

Initially, the inflation bulb valve must be securely closed by rotating it clockwise until fully tightened, thereby preventing premature air leakage. Subsequently, the cuff is wrapped snugly around the upper arm, with careful alignment of the artery marker with the brachial artery. Proper cuff placement is critical for obtaining accurate and reliable readings.

b. Powering On:

The device is activated by pressing the power button once, prompting the system to automatically initialize. If no pressure buildup is detected within 20 s of activation, the device automatically powers down as a protective measure. This ensures that the system is active only during proper use, thereby conserving battery life.

c. Inflation:

Once the system is initialized, the cuff is manually inflated by repeatedly squeezing the inflation bulb. As the pressure increases, the device must be closely monitored. When the target pressure typically between 180 and 200 mmHg is reached, an audible buzzer sounds. This indicates that the optimal pressure for measurement has been achieved. Inflation must be halted immediately upon hearing the buzzer to prevent excessive pressure and potential discomfort.

d. Measurement:

The subject must remain motionless during this phase, as any movement or speech can introduce motion artifacts and measurement errors. The device automatically begins deflating the cuff while simultaneously calculating physiological parameters, including systolic blood pressure, diastolic blood pressure, and heart rate. These values are processed internally and displayed upon completion.

e. Completion:

Upon the conclusion of the measurement, the results are displayed on the device’s screen. To finalize the procedure, the inflation bulb’s valve is opened by turning it counterclockwise. This releases any residual air from the cuff, preparing the device for storage or subsequent use.

### 7.2 Safety hazards and precautions

1. Cuff Placement:

Improper cuff placement can significantly compromise measurement accuracy. An excessively loose cuff may result in erroneous measurements, while an overly tight cuff could cause subject discomfort or restrict blood circulation. Proper sizing and alignment are therefore essential.

2. Over-Inflation Risk:

Exceeding a cuff pressure of 250 mmHg may lead to bruising or numbness in the subject’s arm. Inflation must be halted immediately when the buzzer activates, which indicates that the recommended pressure threshold has been reached.

3. Electrical Safety:

The device must be operated using batteries that are fully charged and correctly oriented. Low battery levels may cause the device to exhibit unpredictable behavior, such as failure to power on or yielding inaccurate readings.

4. Environmental Factors:

External disturbances—such as the subject’s movement, speaking, or muscle tension—can compromise the integrity of the measurement. Measurements must be conducted in a quiet and stable environment to maintain data quality.

5. Device Limitations:

The accuracy of the device depends on periodic calibration, as detailed in Section 5. Calibration protocols must be strictly followed to ensure the long-term reliability of the hardware.

### 7.3 Troubleshooting tips

If the device fails to produce readings, proper connection of the cuff and the correct positioning of the inflation bulb valve must be verified. In the event of inconsistent or unreliable results, recalibration is highly recommended. Additional maintenance guidelines are detailed in Section 5.

#### 7.3.1 Validation and characterization

Prior to validation, the system was tested at individual test points on the PCB, focusing on the signal flow within the signal conditioning section, as illustrated in the block diagram in Fig. 1. The AC component signal, synchronized with the comparator output, is displayed in Fig. 19. Similarly, the synchronized AC and DC components are shown in Fig. 20. Analysis of the comparator output indicates that the heart rate (HR) can be directly measured by counting the pulses generated by this signal. Additionally, both the AC and DC signals exhibit a high signal-to-noise ratio (SNR), ensuring clear and consistent readings. The physical prototype displaying a sample reading and the fully assembled PCB (top and bottom views) are shown in Fig. 21.

**Fig. 19:**
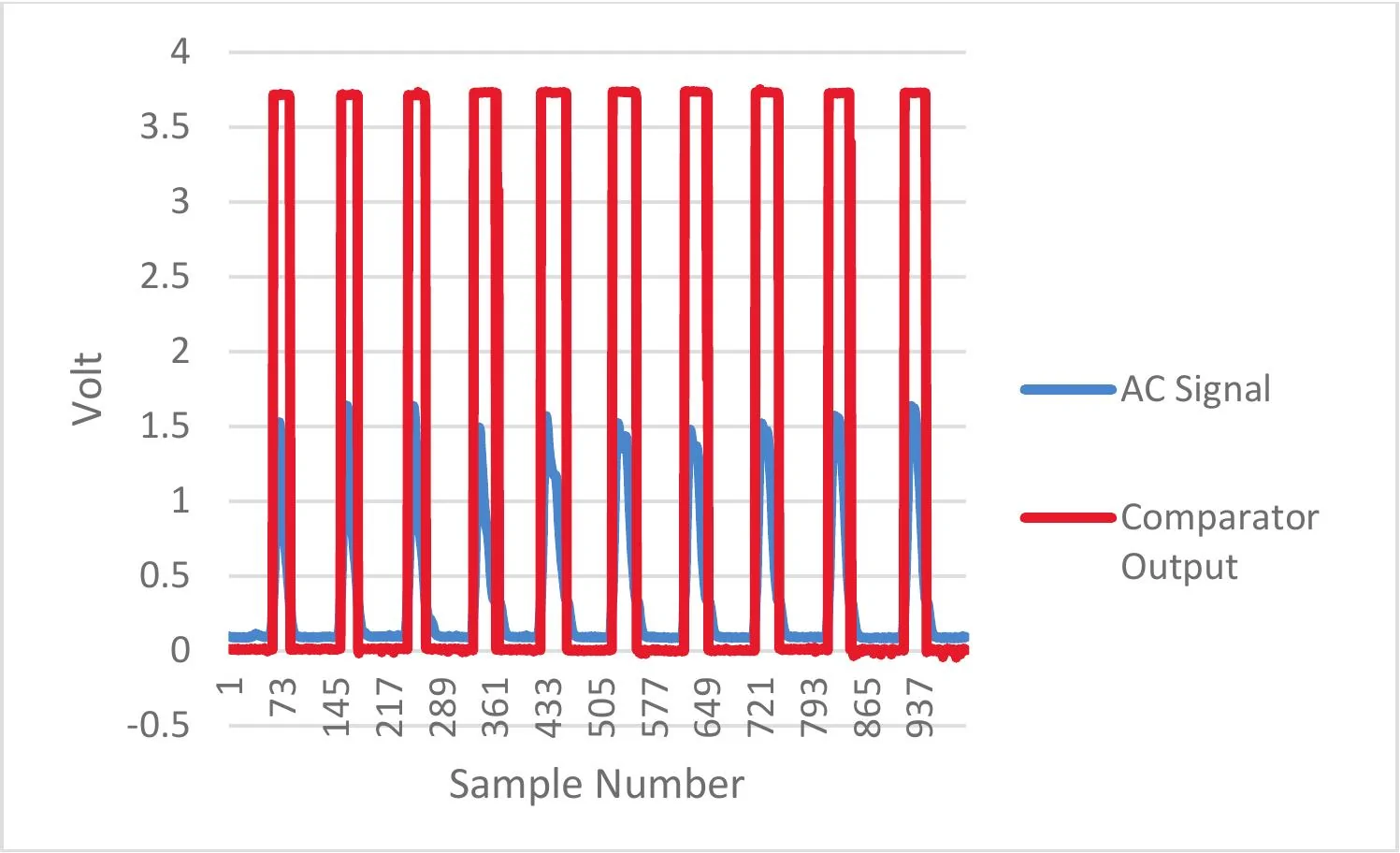
AC components signal with Comparator output signal.

**Fig. 20:**
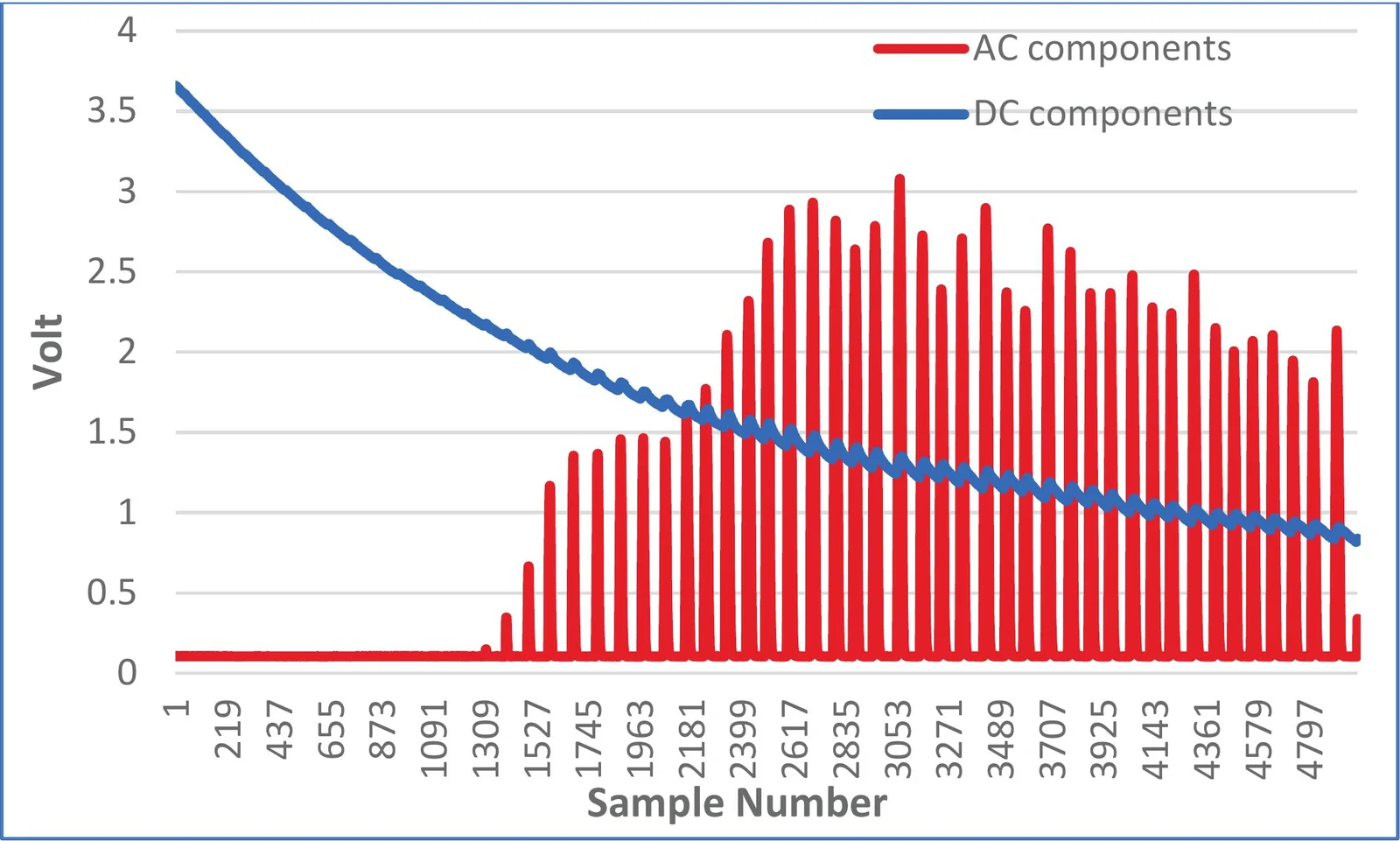
Arterial Pressure Waveform Analysis.

**Fig. 21:**
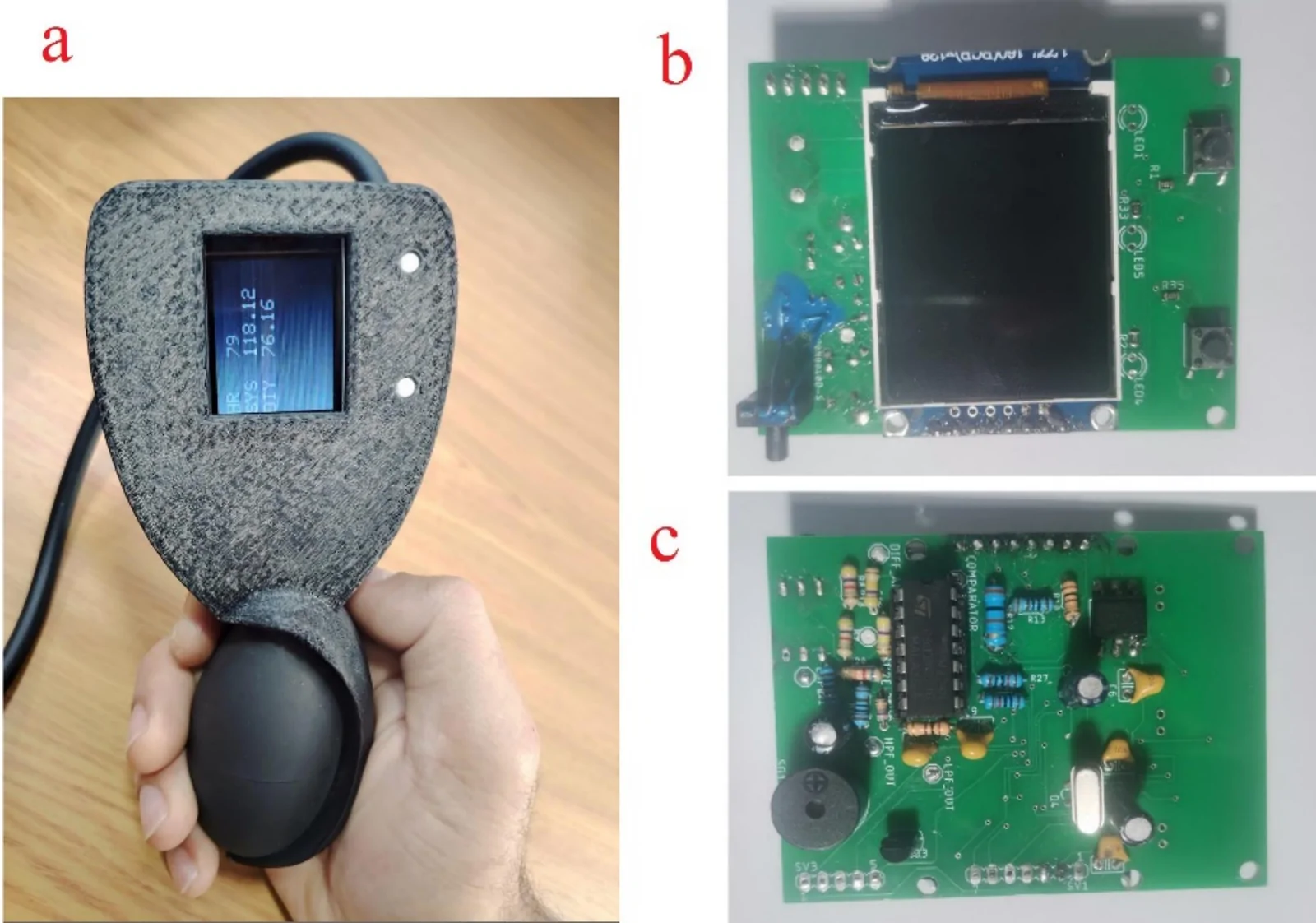
Real-world images for (a) device, (b) Top side PCB, and (c) Bottom side PCB.

The study cohort consisted of 88 adult volunteers with a mean age of 39.4 ± 11.5 years (range: 18–66 years), comprising 47 males (53.4%) and 41 females (46.6%), see Table 3. Blood pressure values spanned a broad clinical range, with systolic pressure ranging from 90.5 to 159.5 mmHg and diastolic pressure ranging from 58.0 to 105.0 mmHg, based on the reference device measurements. Using standard clinical thresholds (SBP ≥ 140 mmHg or DBP ≥ 90 mmHg), 30 participants (34.1%) were classified as hypertensive, while the remaining 58 participants (65.9%) were normotensive. This distribution provided a heterogeneous validation sample encompassing both healthy and elevated blood pressure profiles, supporting a robust assessment of the prototype device’s performance across a clinically representative range of conditions.

**Table 3:** Demographic and clinical characteristics of study participants (N = 88).

Characteristic	Value
Age, years (mean ± SD)	39.4 ± 11.5
Age range	18–66
Male/Female	47/41
SBP range (mmHg)	90.5–159.5
DBP range (mmHg)	58.0–105.0
Hypertensive subjects, n (%)	30 (34.1%)
Normotensive subjects, n (%)	58 (65.9%)

The performance of the prototype blood pressure monitoring device was evaluated through a comparative analysis against two commercially available and clinically validated devices: the Riester and the Microlife. For each of the 88 participants, the prototype recorded three repeated measurements of systolic pressure (SYS), diastolic pressure (DIA), and heart rate (HR), the average of which was compared against a single reference measurement taken with each of the Riester and Microlife devices. The prototype demonstrated strong agreement in systolic pressure and heart rate measurements while showing greater variability in diastolic pressure. The mean differences (± standard deviation) between the prototype and the Riester device were − 1.85 ± 6.74 mmHg for systolic pressure, +4.12 ± 8.13 mmHg for diastolic pressure, and + 3.44 ± 4.23 bpm for heart rate. When compared to the Microlife device, the mean differences were − 3.66 ± 9.90 mmHg for systolic pressure, +3.98 ± 9.82 mmHg for diastolic pressure, and + 5.14 ± 6.38 bpm for heart rate.

Table 4 presents the validation results for a representative sample of the study participants, selected from the full cohort of 88 individuals to illustrate a range of blood pressure cases, including both hypertensive patients and healthy individuals. For brevity, only 12 representative measurements are presented in the main manuscript, while the complete set of measurements for all 88 volunteers is provided in Appendix A.

**Table 4:** Validation results for a representative sample of study participants.

	Prototype			Riester			Micro-life		
Experiment number	SYS	DIA	HR	SYS	DIA	HR	SYS	DIA	HR
1	128	87	80	129	85	80	136	88	70
2	110	61	78	116	66	83	106	50	85
3	142	83	86	145	83	80	143	67	86
4	123	72	80	121	83	75	137	52	69
5	116	97	66	112	96	65	110	98	61
S6	151	85	89	161	70	86	161	100	82
7	109	86	65	118	80	61	118	80	56
8	134	81	85	132	68	83	133	80	89
9	106	62	83	100	55	80	90	62	76
10	113	79	58	122	74	47	126	76	58
11	134	94	85	136	81	79	148	85	76
12	141	88	93	141	88	92	140	87	82

Across the sample, systolic pressure readings from the prototype fell within ±5 mmHg of both reference devices in only 16 of 88 cases (18%), with the largest single deviations reaching −25 mmHg against the Riester device and −28 mmHg against the Micro-life device, likely attributable to signal noise during rapid cuff deflation in individual measurements. Diastolic pressure measurements exhibited greater variability, with deviations reaching up to +23 mmHg against the Riester device and +27 mmHg against the Micro-life device in the most discrepant cases, suggesting the need for potential refinements in the algorithm’s diastolic detection threshold.

Heart rate measurements showed a stronger overall correlation, with differences of ≤5 bpm observed in 32 of 88 cases (36%), though larger discrepancies (up to +16 bpm vs. Riester and +21 bpm vs. Micro-life) were attributed to motion artifacts or irregular pulse waveforms.

Bland-Altman analysis revealed 95% limits of agreement (LOA) for systolic pressure ranging from −12.68 to +8.34 mmHg (Riester, mean bias −2.17 ± 5.36 mmHg) and −21.92 to +15.08 mmHg (Micro-life, mean bias −3.42 ± 9.44 mmHg), and for diastolic pressure ranging from −11.20 to +18.86 mmHg (Riester, mean bias +3.83 ± 7.67 mmHg) and −13.78 to +22.12 mmHg (Micro-life, mean bias +4.17 ± 9.16 mmHg). For reference, the ANSI/AAMI SP10 criteria require a mean difference within ±5 mmHg and a standard deviation no greater than 8 mmHg. The obtained errors are comparable with values reported in the literature; however, the present study should not be interpreted as formal ANSI/AAMI or ISO validation.

Table 5 presents the measurement error comparison between the proposed device and the reference devices. The prototype’s performance is particularly promising given its ultra-low-cost design, though the observed variability in diastolic pressure, and, to a lesser extent, systolic pressure against the Micro-life device, highlights clear opportunities for further refinement. Overall, the prototype demonstrates clinically acceptable accuracy for heart rate and, against the Riester device, systolic pressure measurements, positioning it as a viable solution for low-resource settings, pending minor algorithmic adjustments to improve overall measurement consistency.

**Table 5:** Measurement Error Comparison: Prototype vs. Standard Devices.

Parameter	Prototype vs. Riester		Prototype vs. Micro-life	
	Mean difference	Standard deviation	Mean difference	Standard deviation
Systolic (mmHg)	−2.17	±5.36	−3.42	±9.44
Diastolic (mmHg)	+3.83	±7.67	+4.17	±9.16
Heart Rate (bpm)	3.08	±3.92	+4.83	±6.12

The cost of each component is detailed in Table 2, which shows that the total cost remains under $8. This cost can be significantly reduced through mass production. In large-scale manufacturing, the integration of a customized LCD display specifically designed for blood pressure monitoring could further lower expenses, along with a more cost-effective plastic casing. With these optimizations, the total production cost could be reduced to less than $5 per unit.

To evaluate the clinical impact of the artifact rejection algorithm on blood pressure accuracy, absolute errors for the 12 representative measurements were analyzed. As Table 6 illustrates, disabling the artifact rejection feature significantly compromises accuracy, increasing the Mean Absolute Error (MAE) from 4.50 to 7.33 mmHg for systolic pressure, and almost doubling the error from 6.50 to 12.92 mmHg for diastolic pressure. These findings strongly confirm that the AR algorithm is essential for mitigating motion artifacts and maintaining measurement precision within clinically acceptable limits.

**Table 6:** compares the absolute measurement errors for the 12 representative samples with and without the artifact rejection algorithm.

Measurement	REED without artifact rejection		Error without artifact rejection		Error with artifact rejection	
	SYS	DIA	SYS	DIA	SYS	DIA
1	140	97	11	12	−1	2
2	128	79	12	13	−6	−5
3	154	96	9	13	−3	0
4	129	84	8	1	2	−11
5	128	102	16	6	4	1
6	169	104	8	34	−10	15
7	115	96	−3	16	−9	6
8	134	79	2	11	2	13
9	106	64	6	9	6	7
10	125	87	3	13	−9	5
11	140	100	4	19	−2	13
12	147	96	6	8	0	0
**Mean Absolute Error (MAE)**			**7.33**	**12.92**	**4.50**	**6.50**

### 7.4 Artifact rejection performance

To quantitatively evaluate the artifact rejection algorithm, the number of detected pulses and rejected artifact pulses was recorded for the 12 representative measurements presented in this study. As summarized in Table 7, each measurement contained approximately 40–50 detected oscillometric pulses. The proposed algorithm automatically identified and excluded pulses with abnormal temporal characteristics before constructing the oscillometric envelope used for blood pressure estimation.

**Table 7:** Summary of the artifact rejection performance for the 12 representative measurements.

Measurement	Total detected pulses	Rejected artifact pulses	Accepted pulses	Rejection rate (%)
1	44	4	40	9.1
2	39	3	36	7.7
3	47	5	42	10.6
4	41	1	40	2.4
5	46	4	42	8.7
6	48	7	41	14.6
7	42	2	40	4.8
8	38	0	38	0.0
9	45	1	44	2.2
10	40	2	38	5.0
11	49	3	46	6.1
12	43	2	41	4.7
**Average**	**43.50**	**2.83**	**40.67**	**6.33**

To further evaluate the effect of the artifact rejection procedure on BP estimation accuracy, the absolute errors for the 12 representative measurements were compared with and without artifact rejection. As shown in Table 6, disabling the artifact rejection feature increased the MAE from 4.50 to 7.33 mmHg for SBP and from 6.50 to 12.92 mmHg for DBP. These results indicate that the artifact rejection procedure contributes to reducing measurement errors, particularly for diastolic pressure.

### 7.5 Conclusion

In conclusion, the developed low-cost blood pressure device demonstrates the successful integration of system components to provide user-friendly operation and accurate readings while maintaining affordability. The design eliminates expensive components such as an automated air pump and solenoid valve, opting instead for a single operational amplifier (op-amp) chip and a low-cost display, making the device both economically viable and energy-efficient. Unlike fully automatic systems that consume substantial power for motorized pumps and valves, this device’s energy consumption is significantly reduced, ensuring longer battery life an essential factor for use in low-resource settings, particularly in developing countries. The device’s design is not only functional but also highly accessible, featuring clear guidance messages and a compact, aesthetically pleasing form factor. In terms of accuracy, the results obtained from the prototype are comparable to those of the premium Riester device, and even more precise than those from the Micro-life device. The observed limitations related to accuracy and reading ranges are consistent with those of standard commercially available devices for home or clinic use. This successful prototype provides valuable insights and serves as a platform for future research aimed at refining algorithmic accuracy and reliability, thereby driving further advancements in affordable healthcare technology.

## Ethics statements

This study involved human subjects to validate the developed low-cost blood pressure device. A total of 88 adult volunteers participated in the validation trials. All participants were fully informed about the procedure and provided informed consent prior to their participation. No personally identifiable information was collected, and all data were strictly anonymized. The study was conducted in accordance with established ethical standards for research involving human participants.

## CRediT authorship contribution statement

**Muhammad Al-Ayyad:** Writing – review & editing, Writing – original draft, Conceptualization. **Bashar Al-haj Moh’d:** Supervision, Methodology, Data curation, Conceptualization. **Mohammad Hasan Shamsan:** Visualization, Software, Investigation. **Mohammad Al-Sayed Ahmad:** Validation, Software. **Mohammad Al-Takrouri:** Writing – review & editing, Software. **Hamza Abu Owida:** Writing – review & editing.

## Declaration of competing interest

The authors declare that they have no known competing financial interests or personal relationships that could have appeared to influence the work reported in this paper.

## Figures and Tables

**Table:** 

	**Prototype**			**Riester**			**Micro-life**		
**#**	**SYS**	**DIA**	**HR**	**SYS**	**DIA**	**HR**	**SYS**	**DIA**	**HR**
1	127	83	90	126	81	88	131	95	87
2	107	72	83	113	72	85	123	80	87
3	113	75	80	121	75	85	129	88	79
4	121	86	65	129	87	63	135	91	62
5	139	102	65	138	98	60	141	100	63
6	126	82	85	131	81	74	114	78	77
7	140	90	87	137	70	85	122	73	71
8	149	110	68	144	99	63	147	105	59
9	134	87	67	142	83	70	137	80	64
10	126	87	87	129	78	85	138	63	71
11	136	102	91	135	106	87	155	89	87
12	148	92	69	144	70	65	147	91	73
13	125	77	89	136	83	87	135	62	85
14	125	80	83	130	87	77	142	80	79
15	149	104	87	154	90	87	157	120	98
16	138	94	99	145	83	96	151	99	94
17	122	78	75	111	69	71	105	92	71
18	135	93	70	134	84	64	127	93	60
19	122	83	68	133	79	62	124	79	51
20	122	90	69	118	94	70	107	69	57
21	131	82	77	118	77	79	132	74	73
22	103	69	82	98	72	73	114	67	83
23	106	70	81	119	58	76	91	58	60
24	121	70	87	126	67	86	116	87	81
25	115	81	78	108	84	69	128	74	73
26	132	90	94	139	89	90	136	78	89
27	116	79	75	115	72	67	127	89	68
28	110	73	108	106	75	104	126	58	103
29	147	85	85	156	88	73	139	77	83
30	125	80	69	128	75	58	106	80	66
31	129	83	66	154	77	64	145	73	61
32	109	69	83	119	70	76	105	55	81
33	121	80	76	125	81	69	129	74	74
34	129	89	86	141	84	77	136	83	80
35	113	92	83	104	102	83	121	93	79
36	133	89	77	145	99	71	144	94	62
37	120	83	69	126	86	61	121	71	80
38	124	83	61	126	81	64	135	87	49
39	120	66	73	112	59	74	119	61	60
40	152	98	87	165	82	92	154	99	95
41	128	86	80	122	75	78	132	77	77
42	114	98	64	116	95	58	126	90	61
43	139	90	80	148	86	71	146	76	83
44	112	79	82	111	85	78	105	80	82
45	131	87	68	132	83	58	127	85	70
46	103	62	80	109	61	81	115	67	63
47	111	85	79	112	79	82	111	85	67
48	131	93	65	136	97	62	124	85	52
49	138	97	82	139	89	77	157	86	72
50	130	79	84	128	62	81	126	84	86
51	126	96	90	117	93	94	135	83	88
52	124	72	90	135	65	86	119	55	81
53	109	80	63	96	70	64	119	71	65
54	119	98	68	135	98	62	140	75	58
55	122	74	84	125	69	79	141	78	80
56	142	88	84	140	85	84	143	96	80
57	132	89	84	133	84	82	131	102	74
58	105	69	110	112	72	110	116	55	102
59	132	75	84	136	72	81	127	65	81
60	123	83	90	129	75	83	142	80	78
61	119	72	88	126	56	89	121	65	90
62	136	94	85	132	82	80	151	100	76
63	141	85	75	141	63	73	150	58	65
64	140	104	86	147	108	86	141	99	83
65	117	73	69	113	62	66	128	68	62
66	124	81	75	124	75	76	130	69	78
67	132	94	73	129	71	71	123	86	61
68	141	83	79	138	86	80	148	76	75
69	122	84	103	130	88	92	114	87	101
70	126	95	57	132	96	54	139	86	45
71	114	65	86	110	80	85	109	55	84
72	112	78	60	110	80	56	98	61	63
73	139	93	73	141	97	70	167	74	77
74	146	101	90	147	97	87	156	102	81
75	127	75	79	120	68	73	122	80	81
76	141	82	66	148	62	60	145	79	50
77	133	92	70	131	80	69	130	88	78
78	120	83	85	123	85	84	128	68	69
79	133	90	70	137	94	67	133	102	63
80	148	99	80	142	91	86	151	80	75
81	128	80	79	124	88	81	116	77	76
82	148	98	71	145	79	62	147	98	63
83	95	70	102	88	56	92	93	75	96
84	139	86	85	141	86	82	145	98	73
85	129	101	56	126	113	50	136	89	50
86	124	87	80	129	72	75	130	82	74
87	129	77	71	129	74	55	126	85	67
88	102	76	87	105	62	80	108	85	82

## Appendix A

..
